# Vertical Zonal Distribution Patterns of Entomopathogenic Fungi in the Changbai Mountain

**DOI:** 10.1002/ece3.71623

**Published:** 2025-07-01

**Authors:** Lichao Feng, Kai Yuan, Yan Li, Hang Yang, Songyu Yang, Qingfan Meng

**Affiliations:** ^1^ Forestry College Beihua University Jilin China; ^2^ Guangxi Eco‐Engineering Vocational and Technical College Liuzhou China; ^3^ State Key Laboratory of Soil and Sustainable Agriculture, Institute of Soil Science Chinese Academy of Sciences Nanjing China

**Keywords:** altitude, broad‐leaved forest, shrub diversity, soil physicochemical variables, vegetation belts

## Abstract

Entomopathogenic fungi (EPF) are critical drivers of ecosystem processes such as pest regulation and material cycling. However, their distribution patterns and the drivers influencing them along the elevational gradients remain unclear. This study investigated the diversity and distribution of EPF along a 300–2550 m altitudinal gradient on Changbai Mountain, focusing on their responses to vegetation belts and soil properties. A total of 21 genera and 35 EPF species were identified, with species diversity significantly declining with altitude (Shannon index, *p* < 0.05). High‐altitude communities (1200–2550 m) showed similar structures across coniferous forests, Erman's birch forests, and alpine tundra, whereas low‐altitude communities (300–1050 m) in broad‐leaved and mixed forests were compositionally similar. Narrow‐range species dominated, with many EPF being restricted to specific vegetation belts. Broad‐leaved forests supported the highest EPF diversity, with families such as Cordycipitaceae showing strong preferences for these habitats. Soil properties (e.g., nitrogen, sulfur, phosphorus, moisture content, and pH) and shrub diversity were key drivers of EPF distribution. For instance, the abundance of *Metarhizium*, *Cordyceps*, *Beauveria*, and *Polycephalomyces* was positively correlated with nitrogen and phosphorus but negatively correlated with sulfur. Shrub diversity positively influenced EPF diversity in broad‐leaved forests but negatively in coniferous forests. These findings highlight the interplay between altitude, vegetation, and soil conditions in shaping EPF communities, emphasizing the importance of preserving fungal diversity in alpine ecosystems to maintain ecological balance and support biological control strategies.

## Graphical abstract

Entomopathogenic fungi (EPF) distribution was divided into high‐ and low‐elevation patterns. EPF species diversity in the same vegetation belt was unaffected by altitude. EPF tended to evolve toward broadleaved forest habitats.

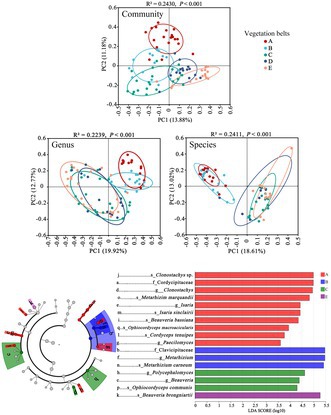

## 1 Introduction

Entomopathogenic fungi (EPF) constitute a group of fungi that can invade the body walls of insects, either proliferating within or producing toxins that can cause the death of the host. This category also encompasses fungal species that are pathogenic to other arthropods such as mites, spiders, centipedes, beetles, and ants (Roy et al. 2006). As key regulators in natural ecosystems, the species diversity and functional differentiation of EPF play a crucial role in host population dynamics and trophic interactions. For instance, *Cordyceps militaris* and *Paecilomyces* spp. have been identified as effective biocontrol agents against lepidopteran and coleopteran hosts (Kryukov et al. 2014). Moreover, their metabolic byproducts can inhibit the growth of competing microbes, thereby indirectly influencing soil microbial communities, including plant pathogens (Moreno‐Gavíra et al. 2020; Lopes et al. 2023). Generalist species such as *Metarhizium carneum* can colonize plant root systems and enhance host plant resistance by inducing systemic immunity (López‐Lima et al. 2023). *Hirsutella thompsonii*, which exhibits specificity toward mites, significantly affects forest litter decomposition, and its chitinase system plays a critical role in carbon cycling (Peng et al. 2002). Similarly, *Beauveria pseudobassiana*, which targets forest pests in coniferous ecosystems, serves as a biological barrier against ecological disasters, such as pine wilt disease (Wang et al. 2020). The spatial divergence of these functional groups likely reflects their coevolutionary adaptation to microenvironmental factors (e.g., climate and host communities), ultimately contributing to the rich diversity of functional guilds (Acheampong et al. 2020; Papudeshi et al. 2023). The diversity advantage of EPF suggests that such ecosystems may maintain higher ecological stability through interactions across multiple trophic levels. However, the stability and diversity of EPF communities are more strongly influenced by habitat conditions shaped by vegetation and the specific ecological niches these environments provide (Vukicevich et al. 2019).

Among these vegetation‐driven habitats, forests are key shapers of terrestrial habitats and providers of essential resources for many organisms, significantly influencing the distribution and composition of entomopathogenic fungal taxa (Hesketh et al. 2010). Along latitudinal gradients, tropical rainforests, characterized by warm, humid climates and year‐round vegetation abundance, exhibit higher diversity and abundance of EPF (Neto et al. 2019). In contrast, temperate deciduous and coniferous forests experience large‐scale infestations of EPF, which are often linked to populations of plant‐feeding insects during the growing season (Pilarska et al. 2006). These fungi also demonstrate pronounced seasonal and regional variability, such as those parasitizing *Ips typographus* in coniferous forests and *Lymantria dispar* in deciduous forests (Kreutz et al. 2004; Pilarska et al. 2006). In boreal coniferous forests, the distribution of EPF is limited to specific taxa, owing to seasonal constraints on host insect activity (Popa et al. 2012). This indicates that latitudinal variation can play a significant role in shaping the composition and distribution of these fungi. Similarly, in subtropical forests, genera such as *Metarhizium* and *Beauveria* exhibit a vertical spatial distribution with altitude, with *Metarhizium* abundance typically decreasing at higher altitudes (Masoudi et al. 2018). Another study in the same climatic zone has suggested that soil fungal abundance can be higher at mid‐altitudes and more homogeneous at lower altitudes (Zheng et al. 2024). These findings confirm that EPF can exhibit distinct ecotypes and geographic distributions across different regions and altitudes (Coates et al. 2002).

The interactions among forest type, vegetation composition, and EPF are complex (McKinnon et al. 2018). EPF can regulate insect populations, thereby affecting forest health and composition (Hallouti et al. 2020). Different forest types and their specific vegetation types can also affect the diversity and abundance of fungi (Deaver et al. 2019). Forests with higher biodiversity support greater insect abundance, which fosters richer entomopathogenic fungal communities (Fisher et al. 2011) and contributes to the high fungal abundance observed in forests. Additionally, the unique climate and microclimate of forest ecosystems, particularly temperature and humidity, can significantly affect fungal communities (Skrzecz et al. 2024). The key catalysts of these microclimates, such as canopy density and vegetation composition, can alter relative humidity levels, thereby altering the mutualistic relationships between insects and fungi (Gandhi et al. 2007). Vegetation composition also determines the quantity and quality of the litter layer, which shapes the soil microenvironment, including moisture, organic matter, and biota, and directly supports the distribution of EPF within litter (Filotas and Hajek 2004; Jaber and Ownley 2018). Therefore, forests profoundly affect the growth and distribution of EPF.

As a natural reservoir of EPF, soil is intricately linked to vegetation composition and diversity and plays a critical role in regulating fungal community composition and distribution (Scheepmaker and Butt 2010). Insects with soil‐based life stages or those foraging for food indirectly influence the fungal distribution (Hesketh et al. 2010). Abiotic conditions, such as soil temperature and water content, directly control the germination and proliferation of fungal spores (Garrido‐Jurado et al. 2011), while soil temperature can affect the rate of fungal colonization (Zembrzuski et al. 2023). The colonization of EPF in seasonally distinct regions exhibits unique geographic distributions and seasonal characteristics, driven by varying sensitivities to temperature (Rangel et al. 2015). In contrast to temperature, humidity plays a more significant role in determining the distribution and spread of fungi. High humidity can enhance fungal growth and spore dispersal, thereby increasing insect infestation and transmission rates (Butt et al. 2016; Islam et al. 2021), whereas dry conditions can limit fungal proliferation and reduce infestation rates (Jackson et al. 2010). Therefore, taxa with higher abundance are typically found in moist soils (Filotas and Hajek 2004), supporting the observation that moist and rainy climates are more conducive to sustaining entomopathogenic fungal populations than drier Mediterranean climates (Rajula et al. 2020).

The soil pH plays a critical role in determining the intensity and distribution of EPF (Padmavathi et al. 2003; Batalla‐Carrera et al. 2013). It influences fungal growth and survival by controlling nutrient solubility and the availability of toxic metals (Abdu et al. 2017; Seguel et al. 2013). Fungal species exhibit varying pH preferences, with some thriving in acidic conditions and others in neutral to slightly alkaline soils (Gleason et al. 2010; Grum‐Grzhimaylo et al. 2016), whereas only a few entomopathogenic fungi have demonstrated broader pH tolerance (Quesada‐Moraga et al. 2007). These preferences can influence the spatial distribution and community composition of EPF in different soil types (Deaver et al. 2019). As essential bulk elements, soil organic matter, nitrogen, and phosphorus play pivotal roles in shaping the community and distribution of EPF. Their abundance directly influences the populations of numerous organisms that either feed on or are indirectly associated with these fungi (Quesada‐Moraga et al. 2007; Kumar et al. 2022). Organic matter also enhances the soil water‐holding capacity and structure (Lal 2020), fostering greater fungal diversity and abundance by providing ample resources and favorable growth conditions (Uzman et al. 2019).

Entomopathogenic fungal taxa exhibit altitudinal variation, with peak abundance at mid‐altitudes and a more uniform distribution at lower elevations (Zheng et al. 2024), whereas relatively stable taxa may persist at different altitudes (Corneo et al. 2013). The global latitudinal patterns of soil fungal taxa suggest that zonal and climatic variability across geographic regions influences the distribution and scale of EPF (Tedersoo et al. 2014). The impact of vegetation type and altitude on fungal community structure should be reassessed by considering the combined effects of vegetation and microhabitat characteristics (Skrzecz et al. 2024). Despite the existing knowledge of the distribution patterns and factors influencing EPF, there is a pressing need for more geographically specific data on their distribution in localized habitats, particularly within altitude‐driven frameworks. Although vegetation is recognized as a key driver of fungal distribution, the complex interactions among vegetation type, soil properties, and fungal adaptation remain poorly understood. To address these gaps, this study investigated the combined effects of these factors on EPF communities across altitudinal gradients, with the aim of providing a comprehensive understanding of the mechanisms shaping fungal diversity and adaptation in diverse ecosystems. We hypothesize that vegetation type is the primary driver of fungal distribution, with adaptive patterns of EPF shaped by vegetation and synergistic interactions with soil physicochemical properties, collectively enhancing species diversity.

## 2 Material and Methods

### 2.1 Overview of the Study Site

The study area is situated on the northern slope of the Changbai Mountain Nature Reserve in southeastern Jilin Province. The reserve spans geographic coordinates 127°42′55″–128°16′48″ E and 41°41′49″–42°25′18″ N, with the main peak reaching an altitude of 2691 m. The region exhibits a distinct vertical distribution of vegetation belts with increasing altitude: broad‐leaved forests at 300–700 m (A), mixed coniferous ( *Pinus koraiensis* ) and broad‐leaved forests at 740–1100 m (B), coniferous forests (including *Abies holophylla*, *Abies nephrolepis*, *Larix olgensis*, and *Picea jezoensis* var. *komarovii*) at (C), Erman's birch (*Betula ermanii*) forests at 1700–2000 m (D), and alpine tundra at 2000–2600 m (E) (Dai et al. 2011).

The study area has diverse soil types, including brown forests, brown coniferous forests, forest meadows, and tundra soils (Jin et al. 2021). Climatic conditions vary significantly across vegetation belts (Hao et al. 2003; Yu et al. 2014). Broad‐leaved forests (A) are characterized by a mean annual temperature of 3°C–5°C, annual precipitation of 800–1000 mm, and relative humidity of 70%–75%. Mixed coniferous and broad‐leaved forests (B) experience a mean annual temperature of 1°C–3°C, annual precipitation of 900–1100 mm, and relative humidity of 75%–80%. Coniferous forests (C) have a mean annual temperature of −1°C to 1°C, annual precipitation of 1000–1200 mm, and relative humidity of 75%–80%. Erman's birch forests (D) exhibit a mean annual temperature of −3°C to −1°C, annual precipitation of 1100–1300 mm, and relative humidity of 75%–80%. Alpine tundra (E) has a mean annual temperature of −7°C to −3°C, annual precipitation of 1200–1400 mm, and relative humidity of 70%–75%.

### 2.2 Sample Plot Setting

Samples were collected from five vertical vegetation belts on Changbai Mountain: broad‐leaved forest, mixed coniferous and broad‐leaved forest, coniferous forest, Erman's birch forest, and alpine tundra from Lushuihe Township to the Changbai Mountain Nature Reserve. Sixteen survey altitudes (300, 450, 600, 750, 900, 1050, 1200, 1350, 1500, 1650, 1800, 1950, 2100, 2250, 2400, and 2550 m) were established from bottom to top, with intervals of 150 m along the contour transects. Each altitude included three sample transects.

### 2.3 Sample Collection and Vegetation Survey

At each altitude sampling transect, a 20 m × 30 m plot was established. Within each plot, a 1 m × 1 m sampling point was selected using the five‐point sampling method. Soil samples were collected at each sampling point using three soil augers with a diameter of 5 cm and a depth of 20 cm, yielding a total soil volume of approximately 1178 cm^3^ per sampling point (calculated as 3 augers × π × (2.5 cm)^2^ × 20 cm). Visible stones and debris were removed from the soil samples before they were placed in sterilized high‐temperature‐resistant polyethylene (PE) bags. For fungal gene sequencing, a composite soil sample was prepared by thoroughly mixing soil from five points in each sampling plot. Approximately 5 g of the mixed soil was transferred into a 5 mL sterile centrifuge tube, labeled, and immediately stored at −80°C until DNA extraction. After each sampling, the soil augers were sterilized with 75% ethanol and rinsed with sterile deionized water to prevent cross‐contamination.

The soil was collected using a soil core sampler (inner diameter: 5 cm; height: 5 cm) within each 20 × 30 m plot to measure physicochemical properties. Tree species and diameter at breast height (DBH) were recorded by the per‐tree checking method (Sumida et al. 2013). Shrub and herbaceous vegetation were assessed using the grid method (Mueller‐Dombois and Ellenberg 1974). Within each plot, five 2 m × 2 m subplots were established to record shrub species and their abundance. For herbaceous vegetation, a 1 m × 1 m subplot was established within each shrub subplot, where all herbaceous plant species were identified, and their abundance was recorded.

### 2.4 Soil Physicochemical Properties

Soil total nitrogen (TN), total carbon (TC), total sulfur (TS), and total hydrogen (TH) were measured using a Vario MACRO cube carbon and nitrogen analyzer (Elementar Analytical Sensing Systems Co. Ltd., Germany). Prior to analysis, soil samples were dried at 60°C and ground to pass through a 0.15 mm sieve to ensure homogeneity. Approximately 20 mg of dried ground sample was placed in a tin capsule and combusted at 1150°C in an oxygen‐rich environment. The resulting gases (e.g., N_2_, CO_2_, SO_2_, and H_2_O) were quantified using a thermal conductivity detector (TCD) and an infrared detector (IR) to determine the concentrations of TN, TC, TS, and TH. Soil total phosphorus (TP) was measured using an inductively coupled plasma‐atomic emission spectrometer (ICP‐AES 9800, Shimadzu, Japan). Prior to analysis, the soil samples were microwave‐digested using a mixture of hydrochloric acid (HCl) and nitric acid (HNO_3_) in a 3:1 volume ratio. The resulting solution was diluted to a known volume with ultrapure water, and TP concentration was determined using ICP‐AES. To ensure analytical accuracy, standard phosphorus solutions were used for calibration, and blank samples were analyzed concurrently to eliminate background interference. Soil pH was measured using a 1:5 soil‐to‐water ratio (*w*/*v*) following the ISO standard 10,390. Bulk density was determined by drying the undisturbed core samples at 105°C overnight and calculating the ratio of dry soil mass to the volume of the core. Soil moisture content was assessed by measuring the weight loss of the samples after drying at 105°C until a constant weight was achieved.

### 2.5 Amplicon Sequencing of Soil Fungi and Screening for Entomopathogenic Fungi

Soil fungal DNA was extracted using a magnetic bead‐based DNA extraction kit (T09‐096; from Shanghai Majorbio Biopharm Technology Co. Ltd.) following the manufacturer's instructions to ensure high‐quality DNA for downstream molecular analysis. The ITS1 and ITS2 primers of fungal rDNA: F: 5′‐CTTGGTCATTTAGAGGAAGTAA‐3′ and R: 5′‐GCTGCGTTCTTCATCGATGC‐3′. PCR amplification was performed in a total reaction volume of 25 μL, containing 2.5 units/μL of high‐fidelity Taq DNA polymerase, 10 mM dNTPs (0.2 mM final concentration for each dNTP), 2× Taq buffer (containing 1.5 mM MgCl₂), 1 μM of each primer (ITS1 and ITS2), and 10 ng/μL of template DNA. The PCR program consisted of an initial denaturation at 95°C for 3 min, followed by 35 cycles of denaturation (95°C for 30 s), annealing (55°C for 30 s), and extension (72°C for 45 s). Final extension was performed at 72°C for 10 min. The amplification products were separated by electrophoresis on 2% agarose gels and purified using an AxyPrep DNA Gel Extraction Kit (Axygen Biosciences, USA). Quantification of the PCR products was performed using QuantiFluor‐ST (Promega Corporation, USA). The purified amplicons were pooled in equal molar amounts and sequenced on the Illumina MiSeq PE250 high‐throughput sequencing platform (Majorbio Biopharm Technology Co. Ltd., Shanghai, China) (Li et al. 2021). Paired‐end reads were merged, quality filtered, and processed based on sequence overlap. Operational taxonomic unit (OTU) clustering and taxonomic classification were performed using the unite8.0/its_fungi database and USEARCH11‐UPARSE algorithm. Taxonomic assignments required a minimum confidence threshold of 0.7, and the OTUs were clustered at 97% sequence similarity.

To ensure accurate analysis and identification of EPF, The Operational Taxonomic Units (OTUs) were identified and organized by taxonomic status before data analysis. Based on the existing literature records of entomopathogenic fungal genera and strains, the target fungi were preliminarily screened, and their corresponding sequencing data were extracted from the original OTU (Sequence Read Archive with accession number PRJNA1245501) dataset (Table S1). EPF species were identified using a phylogenetic tree. Raw sequencing data of EPF from this study for exogenous species of target fungi were downloaded from the NCBI database (https://www.ncbi.nlm.nih.gov/blast/db), and the species with the highest homology were identified using the BLAST tool. The base sequences of the target fungi and acquired strains were combined into a single FASTA file, and a neighbor‐joining phylogenetic tree was constructed using MEGA11 software.

### 2.6 Statistical Analysis

The OTU dataset for EPF screening was normalized using a rarefaction process based on the smallest sample size to mitigate the influence of sample volume on diversity analyses. Pan and core OTU analyses were employed to examine the relationship between soil fungal community diversity and sample collection volume across different vegetation belts and altitude plots, as well as to identify common and unique fungal components in the experimental samples.

The *α*‐diversity indices, including Sobs and Shannon indices, were used to assess the diversity of EPF across different forest types and altitudes. *β*‐Diversity was calculated using the Bray‐Curtis distance algorithm. The Kruskal–Wallis test combined with Dunn's post hoc multiple comparison test (adjusted *p* < 0.05) was used to compare fungal community composition under varying environmental conditions.

One‐way analysis of variance (ANOVA) was conducted to evaluate the effects of elevation and vegetation on the diversity of EPF communities. Post hoc comparisons were performed using Tukey's Honest Significant Difference (HSD) test, with a significance threshold of *p* < 0.05. Multiple linear regression analyses were used to assess the influence of altitude and vegetation belts on EPF diversity. Model goodness‐of‐fit was evaluated using the coefficient of determination (*R* ^2^), and overall model significance was determined using an *F*‐test. The significance of individual predictors was validated using *t*‐tests, also at a significance level of *p* < 0.05. Principal coordinate analysis (PCoA) was used to examine differences in soil fungal and EPF community structures across vegetation belts. The statistical significance of these differences was tested using permutational multivariate analysis of variance (PERMANOVA) based on Bray–Curtis dissimilarity matrices. Additionally, the Adonis test was applied with 999 permutations to further validate the observed differences in community composition, ensuring robust statistical inference.

Venn diagrams were used to compare the species composition of EPF across different vegetation belts. Partial correlation analysis examined the relationships between EPF and trees, shrubs, and herbs within the vertical vegetation structure of the Changbai Mountain. Multilevel species differences in EPF across samples were analyzed using LDA Effect Size (LEfSe). Employing a one‐against‐all comparison strategy, the Kruskal‐Wallis test identified intergroup differences in fungal species abundance, whereas the Wilcoxon rank‐sum test evaluated the consistency of these differences. Linear discriminant analysis (LDA) was used to assess the effect of different species on the group samples. Species with an LDA score ≥ 2 (*p* < 0.05) were considered significant, and LEfSe multilevel species hierarchical tree diagrams were plotted to infer the evolutionary direction of EPF at various taxonomic levels. The significance level was set at *p* < 0.05.

Soil physicochemical properties and vegetation composition factors were standardized using *Z*‐scores. Environmental factors without covariates were identified through multiple covariance tests and variance inflation factor (VIF) analysis. The Bray‐Curtis distance algorithm was used to quantify the community spacing and habitat differences among fungal samples. The Bray‐Curtis distance algorithm and Kruskal–Wallis test with Tukey–Kramer post hoc analysis were used to examine variations in EPF community structure through cluster analysis. Mantel tests and correlation coefficient heatmaps were employed to examine the relationships between soil physicochemical properties, plant community composition, and community structure of EPF.

## 3 Results

### 3.1 EPF Community Composition and Distribution Patterns at Different Altitudes

**FIGURE 1:**
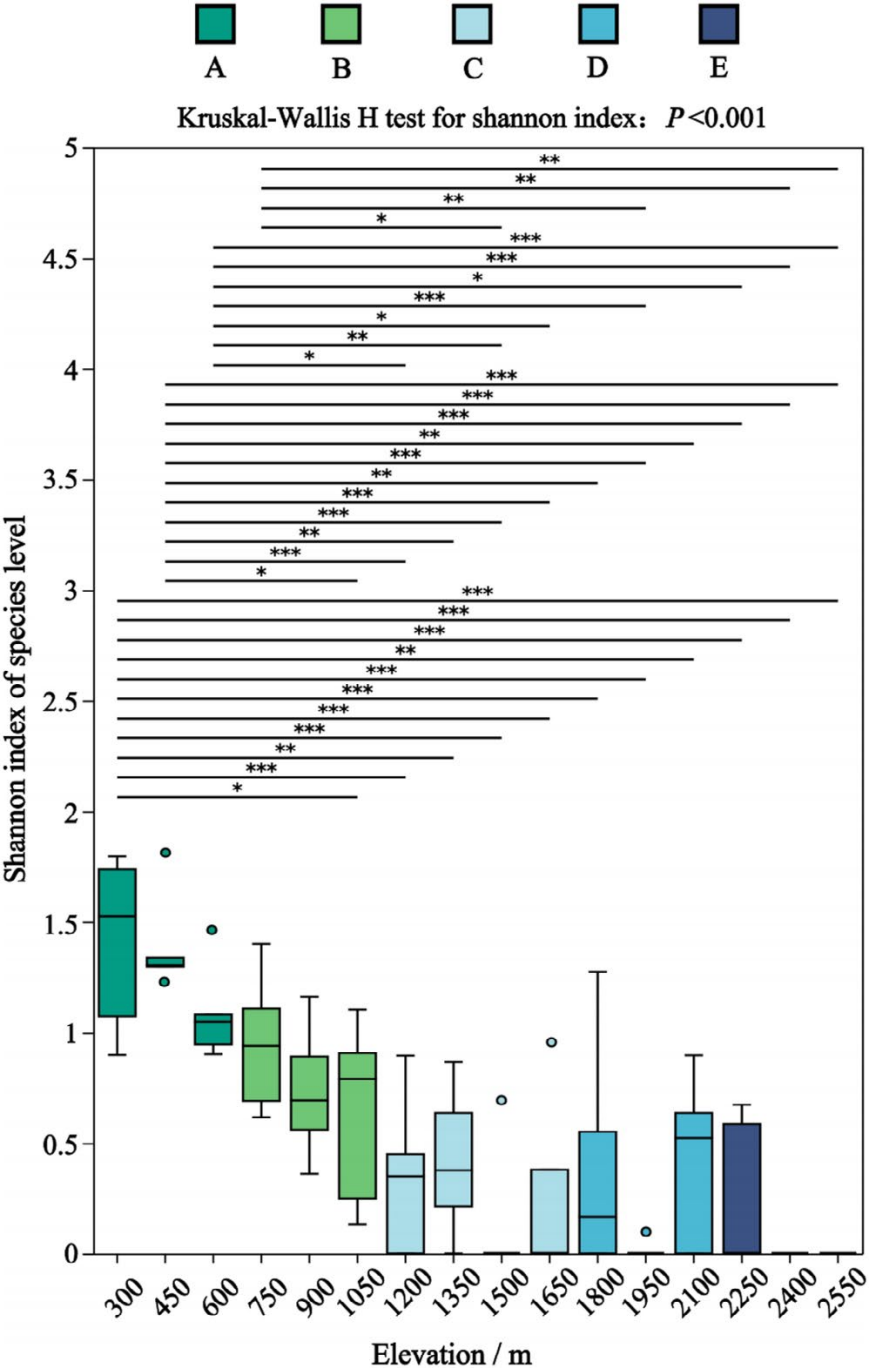
Species *α*‐diversity of EPF at different altitudes. Boxplots of various colors represent different vegetation belts: (A) broad‐leaved forests, (B) mixed coniferous and broad‐leaved forests, (C) coniferous forests, (D) Erman's birch forests, and (E) alpine tundra. The Kruskal–Wallis *H*‐test indicated significant differences at *p* < 0.05. Post hoc analysis using Dunn's pairwise comparisons identified significant differences as follows: *p < 0.05, **p < 0.01, and ***p < 0.001.

**FIGURE 2:**
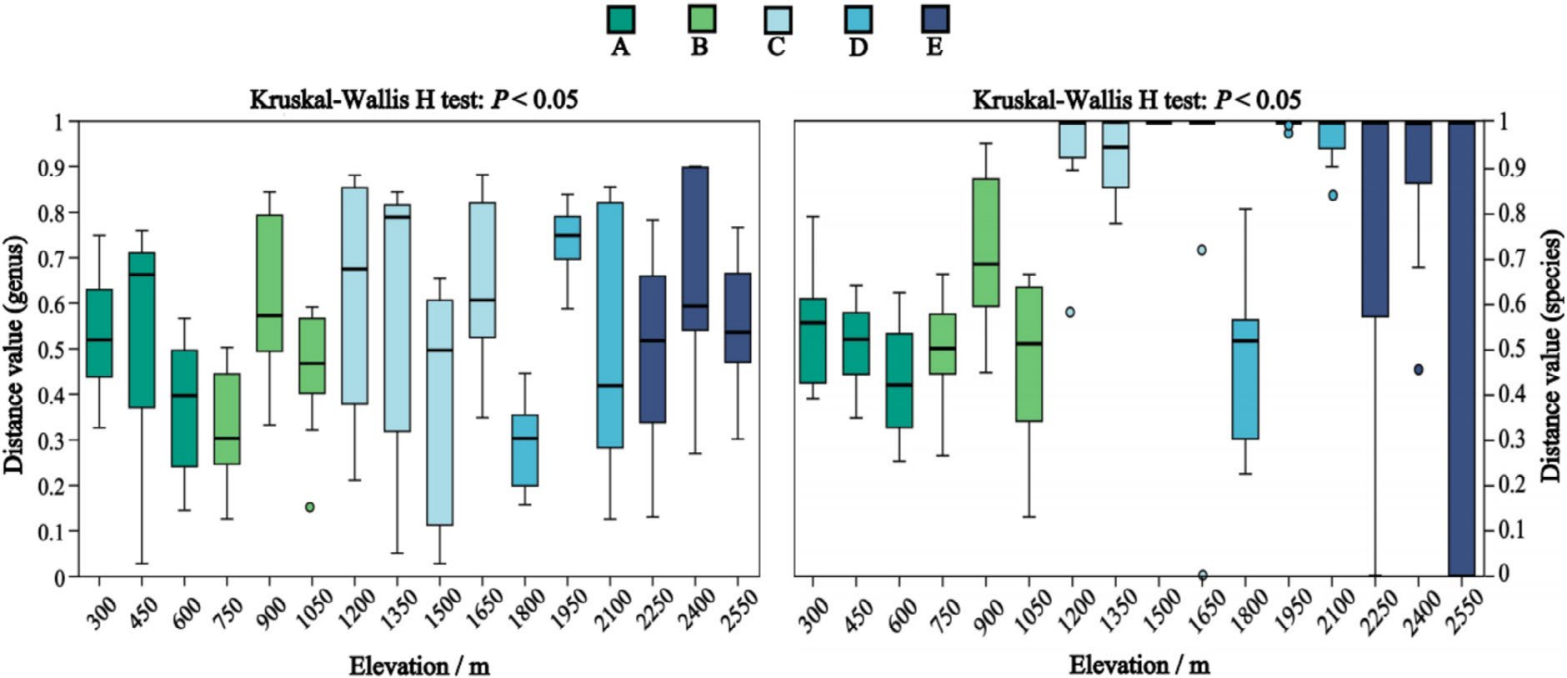
*β*‐Diversity of entomopathogenic fungal genera and species at different altitudes. Boxplots of various colors represent vegetation belts: (A) broad‐leaved forests, (B) mixed coniferous and broad‐leaved forests, (C) coniferous forests, (D) Erman's birch forests, and (E) alpine tundra. The Kruskal–Wallis *H*‐test identified significant differences at *p* < 0.05.

**FIGURE 3:**
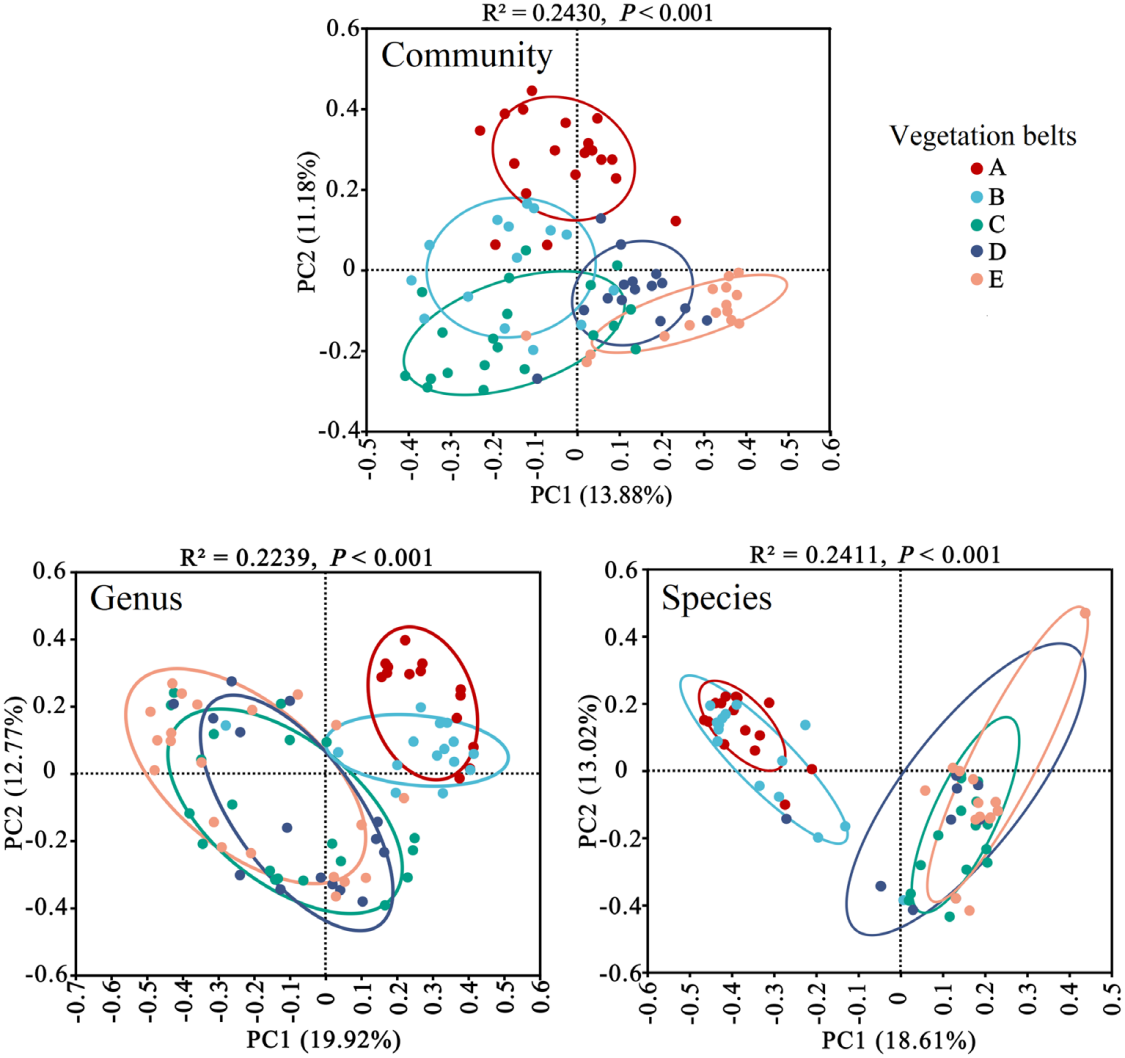
Soil fungal community structure across different vegetation belts. The genus level indicates the genus structure of the EPF community, and the species level indicates the species structure of the EPF community. Vegetation belts are represented by different colors: A‐red (broad‐leaved forests), B‐cyan (mixed coniferous and broad‐leaved forests), C‐green (coniferous forests), D‐blue (Erman's birch forests), and E‐orange (alpine tundra). The *R* ^2^ values indicate the model fit. Significant differences in EPF groupings across vegetation belts were observed (*p* < 0.001).

A total of 21 genera and 35 species of soil EPF were identified on the Changbai Mountain (Figure S1; Table S1). The species diversity (Shannon index) of soil EPF significantly declined with increasing altitude from 300 to 1050 m (Figure 1; Shannon: *p* < 0.05), forming two distinct distribution patterns: high and low altitude (Figures 2 and 3‐Genus and Species). The soil fungal communities exhibited clear altitudinal and vegetation belt distributions (Figure 3‐Community; *R* ^2^ = 0.243, *p* < 0.001; Figure S2). At the genus and species levels, EPF communities in coniferous forests, Erman's birch forests, and alpine tundra (1200–2550 m) demonstrated similar structures. Similarly, the EPF communities in broad‐leaved forests and mixed coniferous broad‐leaved forests (300–1050 m) were similar in composition (Figure 3‐Genus, *R* ^2^ = 0.2239, *p* < 0.001; Figure 3‐Species, *R* ^2^ = 0.2411, *p* < 0.001; Table S2).

At lower altitudes (300–1050 m), both the vegetation belt and altitude had highly significant effects on the diversity and abundance of pathogenic fungi (Table 1, ANOVA, *F* = 26.776, 18.164, 18.161, and 20.018; *p* < 0.0001). At higher altitudes (1200–2550 m), these factors significantly influenced only EPF abundance (Table 1, ANOVA, F = 4.904 and 3.718, *p* < 0.05). When considering the synergistic effects of vegetation type and altitude, altitude and its interaction with vegetation type did not affect EPF diversity. In contrast, vegetation type alone had a significant influence on EPF distribution in low‐altitude areas (Table 2, *p* < 0.001).

**TABLE 1:** Effect of altitude and vegetation belt on EPF communities.

Diversity indices	Low‐altitude (300–1050 m) EPF taxa		High altitude (1200–2550 m) EPF taxa	
	*F*	*p*	*F*	*p*
Shannon (altitude) in community	26.776	< 0.0001	3.985	0.052
Sobs in abundance	18.161	< 0.0001	4.904	0.032
Shannon (vegetation belt) in community	18.164	< 0.0001	1.91	0.161
Sobs in abundance	20.018	< 0.0001	3.718	0.033

**TABLE 2:** Interaction of altitude and vegetation belt on EPF community composition.

Indices & variant	Low‐altitude (300–1050 m) EPF taxa		High altitude (1200–2550 m) EPF taxa	
	Shannon		Shannon	
	*R* ^2^ = 0.403, *p* = 0.003		*R* ^2^ = 0.102, *p* = 0.152	
	*F*	*p*	*F*	*p*
Intercept	299.643	< 0.0001	21.2	< 0.0001
Vegetation belts	19.124	< 0.0001	2.043	0.144
Different altitude in the same vegetation belt	2.398	0.112	1.377	0.265
Vegetation belts × different altitude in the same vegetation belt	0.342	0.714	1.542	0.211

### 3.2 EPF Distribution Trends Across Vegetation Belts

**FIGURE 4:**
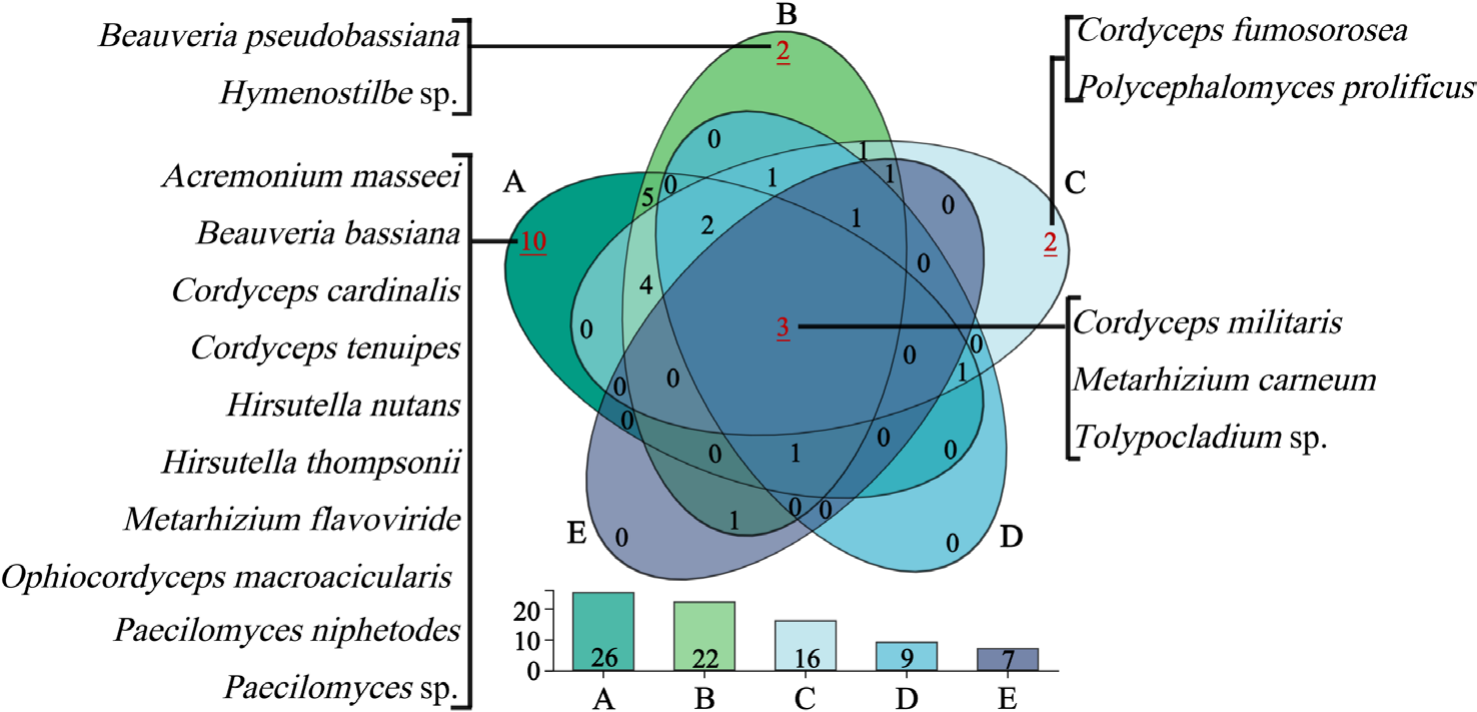
Species composition and distribution of EPF in different vegetation belts. Colors represent different vegetation belts: (A) broad‐leaved forests, (B) mixed coniferous and broad‐leaved forests, (C) coniferous forests, (D) Erman's birch forests, and (E) alpine tundra. The numbers in the ellipses indicate the number of exclusive and shared species in the different vegetation belts, illustrating species composition.

**FIGURE 5:**
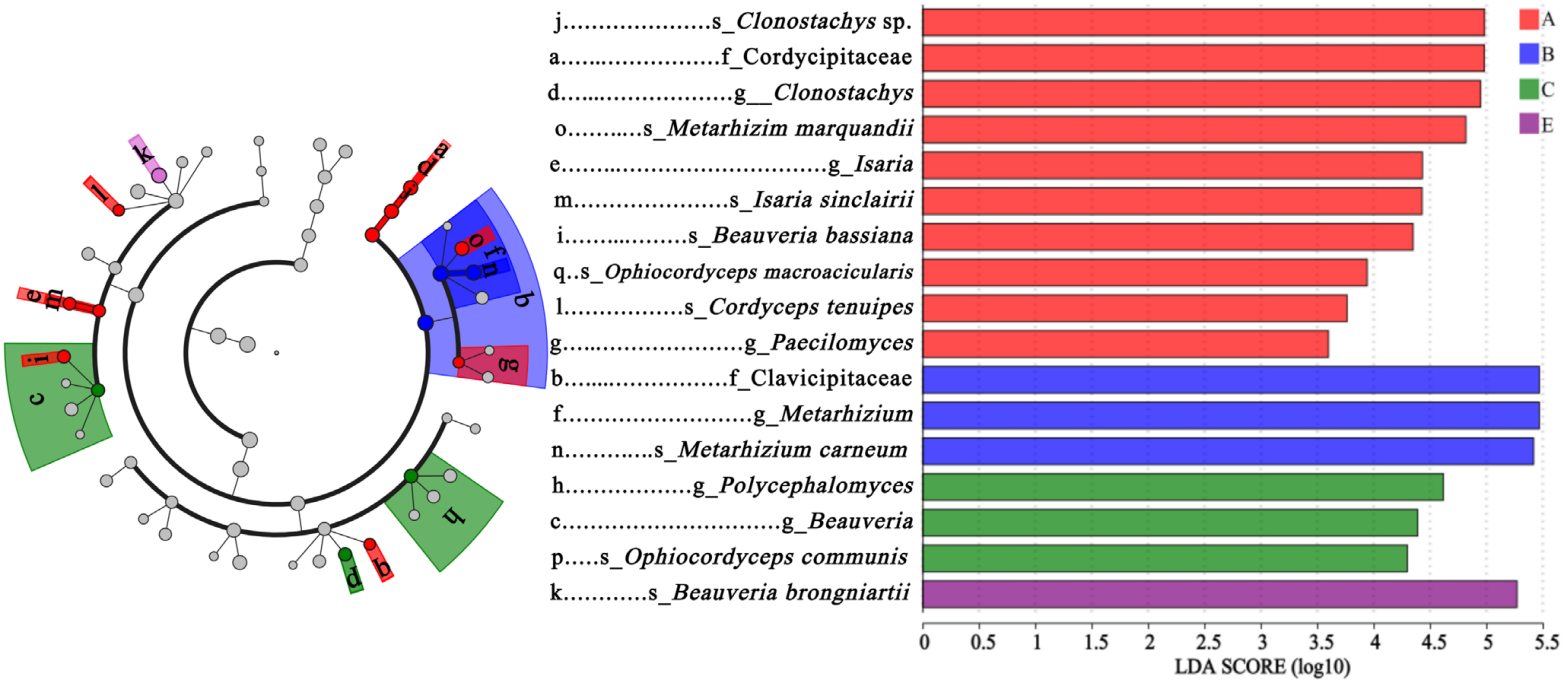
Dominant species and evolutionary trends of EPF in vegetation belts. Concentric circles (inner to outer) represent taxonomic levels from phylum to species, with circle size proportional to abundance. Colors depict species variation across vegetation belts: Gray (no difference), red (broad‐leaved forests), blue (mixed coniferous and broad‐leaved forests), green (coniferous forests), and purple (alpine tundra). Letters denote species differing by vegetation belts, labeled on the right. Distribution histograms highlight species with LDA scores > 2. Bar colors correspond to vegetation belts, and bar lengths indicate the LDA scores, representing the degree of responsibility for significant differentiation.

Among the different vegetation belts, only *Cordyceps militaris*, *Metarhizium carneum*, and *Tolypocladium* sp. were the widespread species. The narrow‐range species were dominant, with *Cordyceps fumosorosea* and *Polycephalomyces prolificus* identified exclusively in coniferous forests; *Beauveria pseudobassiana* and *Hymenostilbe* sp. in the mixed coniferous and broad‐leaved forests; and *Acremonium masseei*, *B. bassiana*, *C. cardinalis* , *C. tenuipes* , *Hirsutella nutans*, *H. thompsonii*, *Metarhizium flavoviride*, *Ophiocordyceps macroacicularis*, *Paecilomyces niphetodes*, and *Paecilomyces* sp. restricted to broad‐leaved forests (Figure 4). Cordycipitaceae demonstrated a preference for broad‐leaved forests, Clavicipitaceae for mixed coniferous and broad‐leaved forests, and certain Paecilomyces and *Metarhizium* species favored broad‐leaved forests. *Beauveria* and Polycephalomyces are more common in coniferous forests (Figure 5). These findings highlight that the broad‐leaved forest habitat at lower altitudes in Changbai Mountain supports greater EPF diversity, with a clear adaptation of EPF to this environment.

### 3.3 Effects of Soil Properties and Shrub Diversity on EPF Distribution

**FIGURE 6:**
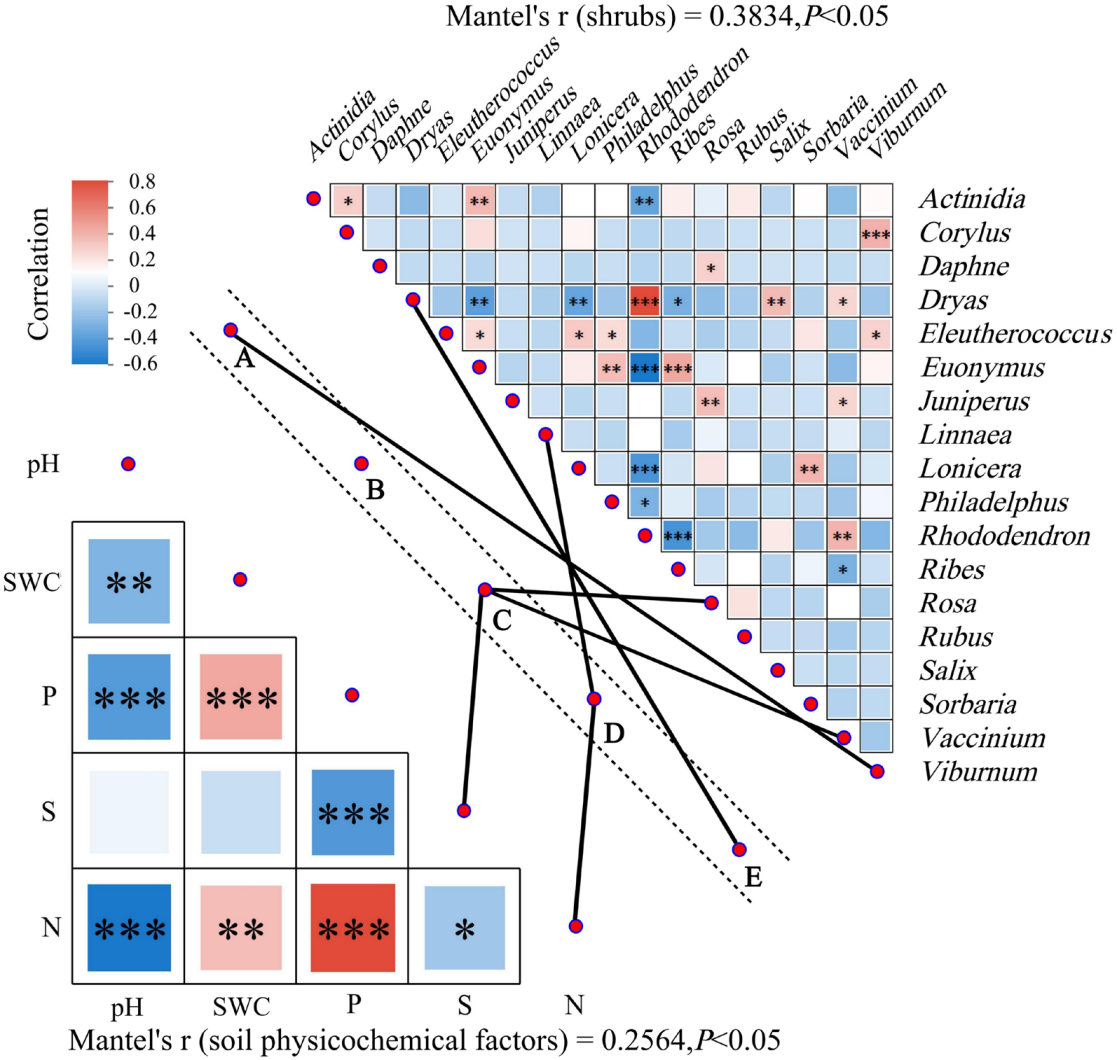
Effects of soil environmental factors and shrub communities on EPF structure across vegetation belts. The lower‐left graph shows correlations between soil physicochemical properties and vegetation belts, and the upper‐right graph shows correlations between shrub taxa. Pearson's *r* test was used to assess correlations, with red indicating positive correlations, blue indicating negative correlations, and significance levels represented as *p < 0.05, **p < 0.01, and ***p < 0.001. The correlation strength between entomopathogenic fungal communities and soil physicochemical properties or shrub species in vegetation belts (A: Broad‐leaved forests; B: Mixed coniferous and broad‐leaved forests; C: Coniferous forests; D: Erman's birch forests; and E: Alpine tundra) is shown by black connecting lines. Thick lines represent moderate correlations (Mantel's *R* = 0.4–0.6), and thin lines indicate weaker correlations (*R* < 0.4), with all correlations significant at *p* < 0.05.

**FIGURE 7:**
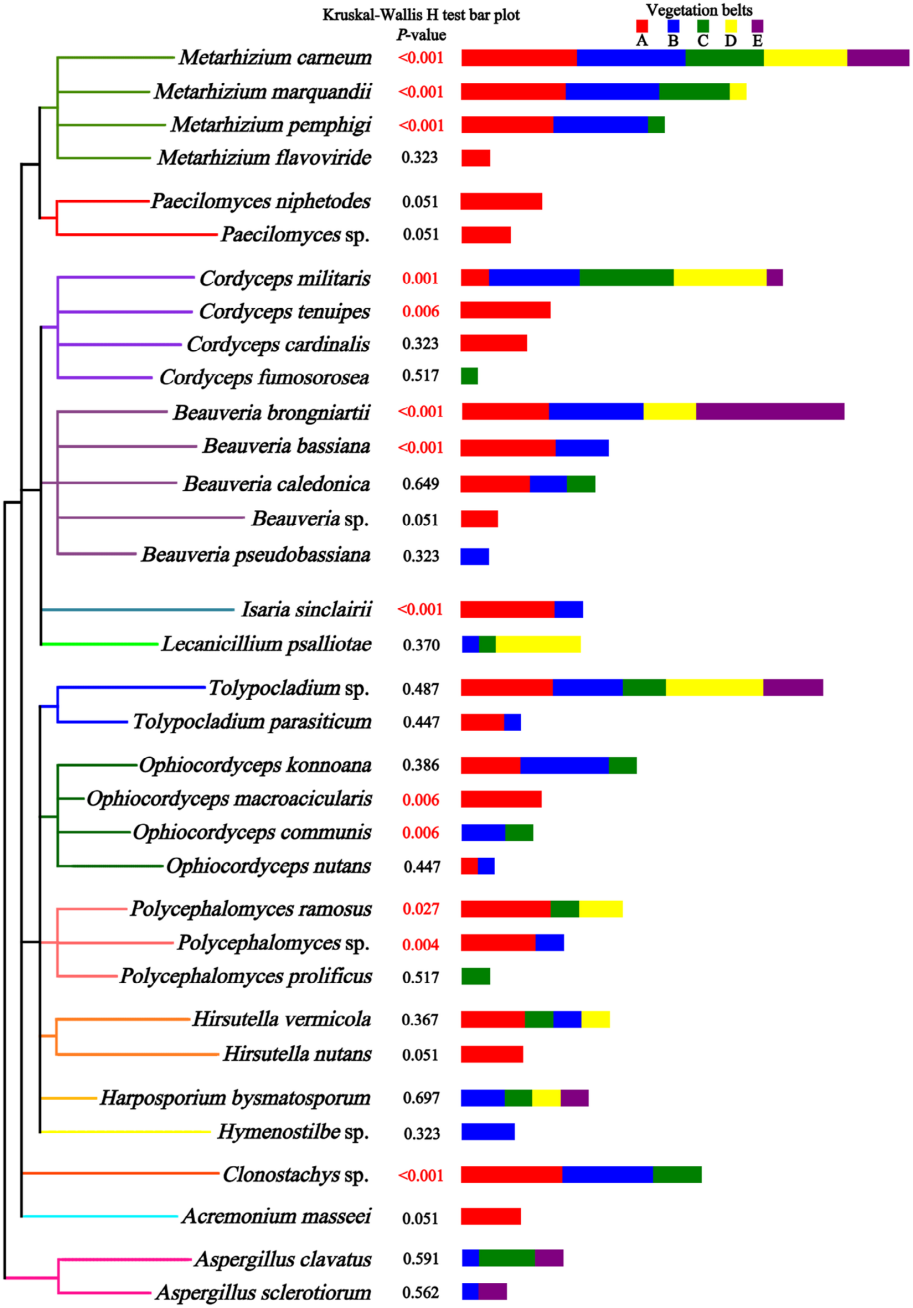
Intergroup distribution differences in EPF across vegetation belts. The clustering tree (right) uses branch colors to indicate EPF genera. The stacked bar chart (left) displays the relative abundance of EPF species across vegetation belts: (A) broad‐leaved forests, (B) mixed coniferous and broad‐leaved forests, (C) coniferous forests, (D) Erman's birch forests, and (E) alpine tundra. Differences in EPF abundance between vegetation belts were evaluated using the Kruskal‐Wallis *H* test. Species with significant differences (*p* < 0.05) are marked with red numbers.

**FIGURE 8:**
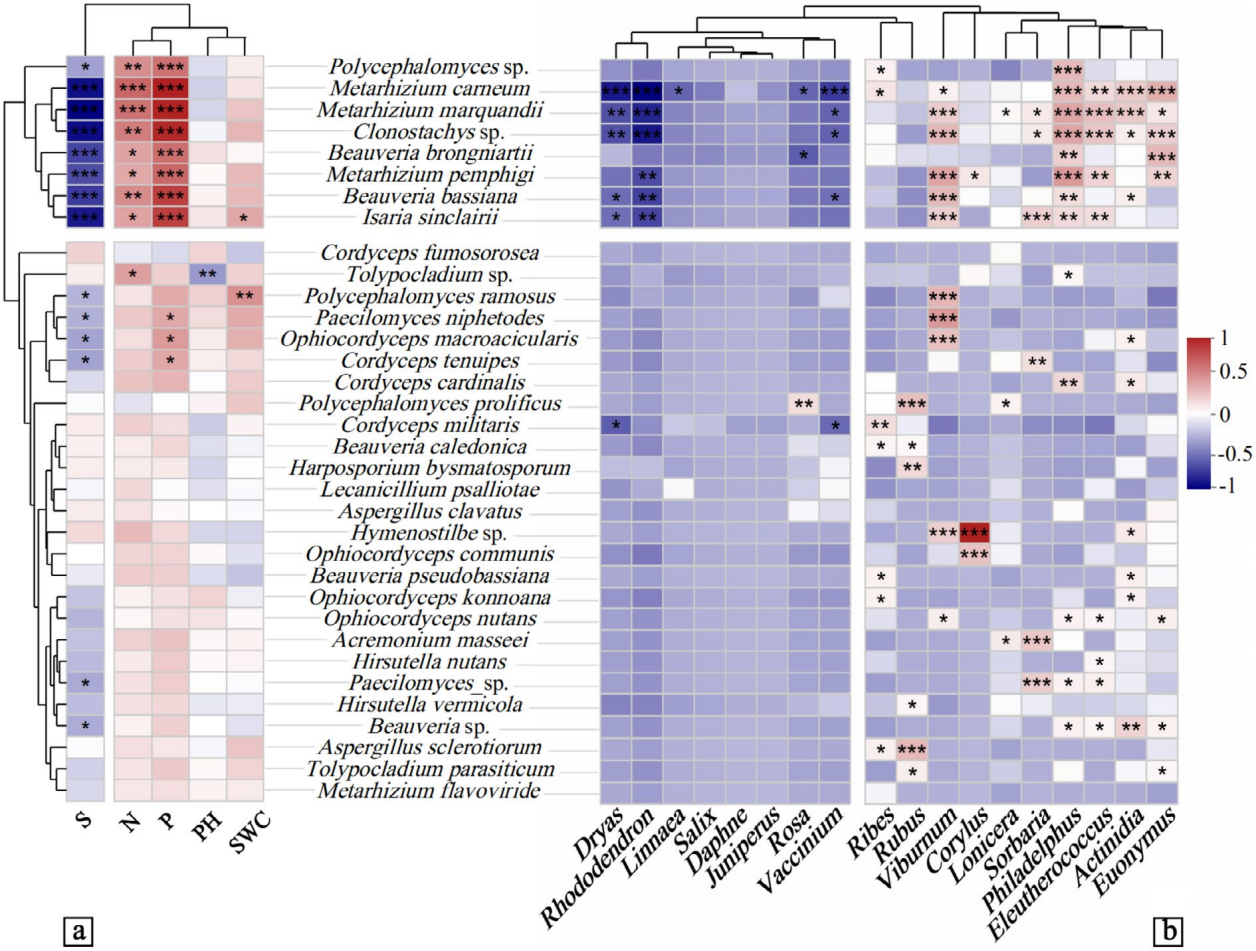
Correlation coefficients of soil physicochemical factors and shrub diversity with EPF. Pearson's *r* test was used to assess correlations between EPF, environmental factors, and shrub taxa. Positive correlations are shown in red, and negative correlations are shown in blue, with significance levels denoted as *p < 0.05, **p < 0.01, and ***p < 0.001.

Soil nitrogen (N), sulfur (S), phosphorus (P), soil water content (SWC), pH, and shrub diversity were identified as key factors influencing the diversity and distribution of EPF based on the physicochemical properties of the soil (Table 3). Soil total nitrogen, moisture content, and shrub diversity significantly affected EPF diversity (Figure 6; soil physicochemical factors: Mantel's *r* = 0.2060, *p* < 0.001; shrub diversity: Mantel's *r* = 0.3757, *p* < 0.001). Thirteen EPF species from the genera *Metarhizium*, *Cordyceps*, and *Polycephalomyces* exhibited significant distributional differences across different vegetation belts (Figure 7; *p* < 0.05). Among the vegetation belts, only shrub diversity showed a significant positive correlation with EPF (Table 4; *p* < 0.05), whereas tree and herbaceous diversity had relatively weak effects on EPF. Specifically, the diversity of EPF species such as *Metarhizium*, *Cordyceps*, *Clonostachys* sp., and *Isaria sinclairii* was negatively correlated with shrub diversity in coniferous forests (e.g., *Rosa* and *Vaccinium*), Erman's birch forests (e.g., *Linnaea*), and alpine tundra (e.g., *Dryas*). However, these species were positively correlated with shrub diversity in broadleaf forests (e.g., *Viburnum*, *Philadelphus*, *Eleutherococcus*, *Actinidia*, and *Euonymus*) (Figure 8; *p* < 0.05). Furthermore, phosphorus and nitrogen were positively correlated with *Metarhizium*, *Cordyceps*, *Beauveria*, *Clonostachys* sp., and *Polycephalomyces* sp., whereas sulfur exhibited a significant negative correlation with these EPF (Figure 8; *p* < 0.05).

**TABLE 3:** Selection of VIF variance for soil environmental factors.

Filtering	VIF values for soil physicochemical factors							
	N	C	H	S	P	SWC	BD	pH
Pre‐filtering	35.8339	34.9890	12.6428	1.9546	5.4267	3.3575	2.3928	1.4185
Post‐filtering	3.5976	—	—	1.9049	3.4120	2.5181	—	1.2960

Abbreviations: BD, soil bulk density; C, total soil carbon content; H, total soil hydrogen content; N, total soil nitrogen content; P, total soil phosphorus content; pH, soil hydrogen ion concentration; S, total soil sulfur content; SWC, soil water content.

**TABLE 4:** Partial correlation of vertical structure of vegetation with diversity of EPF in different vegetation belts.

Control variables	Explanatory variables	EPF		Trees		Shrubs		Herb	
		DP	*p*	DP	*p*	DP	*p*	DP	*p*
—	EPF	1	—	0.462	0.131	0.573	0.052	−0.005	0.989
	Trees			1	—	0.728	0.007	0.375	0.229
	Shrubs					1	—	0.446	0.146
	Herbs							1	—
Herbs	EPF	1	—	0.5	0.117	0.643	0.033		
	Trees			1	—	0.676	0.022		
	Shrubs					1	—		
Shrubs	EPF	1	—	0.079	0.818			−0.355	0.284
	Herbs			0.082	0.81			1	—
	Trees			1	—				
Trees	EPF	1	—			0.389	0.236	−0.216	0.523
	Shrubs					1	—	0.272	0.418
	Herbs							1	—
Trees & shrubs	Herbs	−0.364	0.302					1	—
Herbs & trees	Shrubs	0.477	0.163			1	—		
Herbs & shrubs	Trees	0.116	0.75	1	—				

*Note:* DP was the coefficient of partial correlation, *p* was the significance of partial correlation (two‐sided).

## 4 Discussion

### 4.1 Spatial Patterns and Influencing Factors of EPF Diversity

This study revealed a clear vertical distribution pattern of EPF communities on Changbai Mountain, characterized by a decline in species diversity with increasing altitude. Notably, this altitudinal pattern resulted in the formation of two distinct EPF community assemblages, one at high elevations and the other at low elevations, with an approximate boundary of 1100 m. This variation in diversity reflects the combined effects of environmental gradients, such as shifts in temperature and humidity, as well as the differentiation of vegetation zones (Wang et al. 2016; Masoudi et al. 2018). Moreover, our findings indicated that although altitude and its interaction with vegetation belts contributed to EPF distribution, vegetation type emerged as the primary driver. When the effect of altitude is controlled by vegetation type, its independent influence becomes negligible, reinforcing the dominant role of vegetation composition in shaping EPF spatial patterns (Chen, Chen, et al. 2019). This result not only aligns with global findings on EPF species dynamics (Deaver et al. 2019) but also underscores the importance of regional ecological factors and community characteristics in refining broader ecological patterns.

Vegetation composition is a key driver of EPF diversity and community structure in the Changbai Mountain. EPF exhibits a marked preference for broad‐leaved forest habitats, driven by mutualistic interactions, environmental carrying capacity, and resource availability (McKinnon et al. 2018; Chen, Huang, et al. 2019). Specifically, the higher temperature and humidity in low‐elevation broad‐leaved forests and mixed forests create favorable environments for thermophilic and moisture‐loving fungal taxa such as *Clonostachys* and *Metarhizium* (Cota et al. 2008; Geml et al. 2014). In contrast, EPF diversity significantly declined in high‐elevation coniferous forests and alpine tundra ecosystems, forming a striking contrast to the lowlands.

Notably, the dominant shrub species within different vegetation zones play a crucial role in shaping the regional distribution of EPF by providing specific microhabitats. For example, shrubs such as *Schisandra* and *Viburnum* in broad‐leaved forests, *Vaccinium* in coniferous forests, and *Cassiope* in alpine tundra significantly affect EPF niches by influencing litter decomposition, soil structure, and microbial community characteristics (Chroňáková et al. 2019; Amaral et al. 2022). Our research further showed that shrub diversity in broad‐leaved forests (e.g., plants from the genera *Aralia* and *Euonymus*) significantly promoted EPF diversity, whereas dominant shrubs in high‐elevation habitats (e.g., *Vaccinium* and *Cassiope*) often had the opposite effect. These findings revealed positive and negative correlations between shrub species and EPF distribution, indicating that the distinct characteristics of vegetation types and associated microhabitats not only directly regulate EPF distribution patterns but may also enhance their adaption to specific regions (Neto et al. 2019). This underscores the need for further investigation of the regional adaptive traits of EPF.

Although vegetation zones are the primary determinants of EPF distribution, the role of insect hosts and the ecological niche of EPF should not be overlooked. While this study did not directly assess the impact of insect host populations, existing research indicates that host insect species and densities significantly influence EPF spatial distribution (Hesketh et al. 2010). Evidence from other regions suggests local coevolutionary adaptations between EPF and their insect hosts (Zhang et al. 2014; Naranjo‐Ortiz and Gabaldón 2019). For example, the widespread distribution of *Metarhizium* and *Beauveria* may be closely linked to the diversity and abundance of insect communities (e.g., Coleoptera and Lepidoptera) in low‐elevation regions (Kryukov et al. 2014; Ceryngier 2000). Conversely, certain EPF (e.g., species in the genera *Cordyceps* and *Polycephalomyces*) limited to high‐altitude coniferous forests may adapt to the specific traits of insect populations in these habitats (Zha et al. 2021; Wang et al. 2022).

Variations in insect species composition across different elevations and latitudes may further enhance the spatial differentiation of EPF. Geographic factors, such as elevation, longitude, and latitude, exert a significant influence on EPF community composition and distribution patterns (Wakil et al. 2014). For instance, even within the same soil ecosystem at a given site, EPF can exhibit considerable genetic diversity and occupy distinct ecological niches driven largely by variations in host insect populations (Cabrera‐Mora et al. 2019). Further investigation into the ecological interactions between insect hosts and EPF, particularly in the context of insect migration and the impact of global warming, will be essential for advancing our understanding of EPF ecological adaptability.

### 4.2 Mechanisms of Synergistic Effects Between Biotic and Abiotic Factors

Soil environments play a pivotal role in regulating EPF community composition and diversity, although their effects vary between regions and fungal groups (Peng et al. 2019; Hallouti et al. 2020). This study found that different EPF taxa exhibited varying degrees of dependence on soil physicochemical properties. For example, genera such as *Alternaria* and *Harposporium* demonstrate high environmental adaptability because of their broad tolerance to soil factors (Quesada‐Moraga et al. 2007). In contrast, *Beauveria* and *Metarhizium* showed significant positive correlations with soil nitrogen (N) and phosphorus (P) levels but were negatively correlated with sulfur (S), indicating a direct and significant influence of soil nutrient content on certain EPF distributions (Scheepmaker and Butt 2010; Moloinyane et al. 2020). Additionally, soil texture and structure greatly affect spore dispersal and colonization. For instance, EPF in broad‐leaved forests is limited by soil water content (SWC), whereas EPF in coniferous forests is primarily regulated by pH levels. These differences suggest that soil moisture and acidity exert divergent physiological and metabolic demands on different fungal taxa (Garrido‐Jurado et al. 2011; Hartmann and Six 2023).

Our findings highlight the synergistic effects of vegetation structure and soil properties in shaping the EPF communities. In low‐elevation broad‐leaved forests, higher soil nutrient levels (e.g., N and P) and greater shrub diversity enhance the soil microbial environment and accelerate plant litter decomposition, thereby promoting higher EPF diversity. In contrast, high‐elevation regions are subject to environmental constraints due to soil deficiencies in essential elements such as iron and potassium, which limit EPF diversity (Tedersoo et al. 2014; Zhou et al. 2024). Therefore, these regional differences in soil and vegetation characteristics jointly influence EPF distribution through both direct and indirect mechanisms.

Beyond the roles of vegetation and soil, EPF communities are also regulated by interactions with other soil microorganisms, including bacteria and fungi, via mechanisms such as competition and symbiosis (Nazir et al. 2009; Raja et al. 2021). These multispecies interactions significantly shape EPF ecological niches and survival strategies (Mayerhofer et al. 2017). Moreover, studies have shown that EPF associated with generalist insect hosts tends to exhibit broader distributions, whereas those specializing in particular host species are often limited by host availability (Przybył et al. 2008; Qiu et al. 2023).

## 5 Conclusion

The community structure and distribution of EPF in Changbai Mountain were jointly influenced by altitude and vegetation belts. Within the high‐ and low‐altitude communities formed by altitudinal segmentation, the impact of altitude within the same vegetation belt was significantly diminished, resulting in a bidirectional adaptive relationship between vegetation belts and EPF. Altitude provided a general framework for EPF vertical distribution, with vegetation belts serving as fundamental units and shrubs and soil acting as core components influencing EPF distribution within these units. This study provides evidence that altitude‐driven spatial distribution patterns of EPF exhibit distinct geographical zonal characteristics, offering insights into their distribution and evolutionary trends across geographic regions. However, our understanding of how biotic interactions shape EPF distribution remains limited because of the absence of host insect sampling and the exclusion of transitional zones between vegetation belts from the analysis. Future research should aim to investigate the distribution patterns of insect‐pathogenic fungi in greater detail, including the specialization and generalization of insect‐pathogenic microbial communities, their interactions with insect hosts, and their coevolutionary dynamics. Long‐term, large‐scale monitoring is also essential for assessing EPF responses to climate and land‐use changes. Moreover, the integration of omics technologies such as genomics and transcriptomics will offer deeper insights into the physiological and functional adaptations of EPF to diverse environmental conditions.

## Author Contributions

**Lichao Feng:** conceptualization (equal), investigation (equal), methodology (equal), project administration (equal), resources (equal), software (equal), supervision (equal), validation (equal), visualization (equal), writing – original draft (equal). **Kai Yuan:** formal analysis (equal), investigation (equal), methodology (equal), software (equal). **Yan Li:** investigation (equal), methodology (equal). **Hang Yang:** investigation (equal), methodology (equal), resources (equal). **Songyu Yang:** conceptualization (equal), validation (equal), visualization (equal), writing – review and editing (equal). **Qingfan Meng:** conceptualization (equal), funding acquisition (equal), supervision (equal), validation (equal), visualization (equal), writing – review and editing (equal).

## Conflicts of Interest

The authors declare no conflicts of interest.

## Supporting information


**Figure S1.** Phylogenetic tree of EPF from Changbai Mountain.

**Figure S2.** Pan_OTU and Core_OTU statistics of EPF communities across different forest belts and altitudes.

**Table S1.** Species composition of EPF in Changbai Mountain. Note: Species identified as EPF are referenced from relevant publications. An asterisk (*) indicates that when the number of unidentified species in a genus equals 0, the genus is considered absent in Changbai Mountain and is excluded from subsequent analysis.

**Table S2.** *T*‐test analysis of diversity index of entomopathogenic fungi at different altitudes.

## Data Availability

Data will be made available on request, and can be accessed at https://doi.org/10.5061/dryad.qnk98sft0.

## References

[ece371623-bib-0001] Abdu, N. , A. A. Abdullahi , and A. Abdulkadir . 2017. “Heavy Metals and Soil Microbes.” Environmental Chemistry Letters 15: 65–84.

[ece371623-bib-0002] Acheampong, M. A. , C. A. Coombes , S. D. Moore , and M. P. Hill . 2020. “Temperature Tolerance and Humidity Requirements of Select Entomopathogenic Fungal Isolates for Future Use in Citrus IPM Programmes.” Journal of Invertebrate Pathology 174: 107436. 32619548 10.1016/j.jip.2020.107436

[ece371623-bib-0003] Amaral, A. G. , N. R. Bijos , P. Moser , and C. B. R. Munhoz . 2022. “Spatially Structured Soil Properties and Climate Explain Distribution Patterns of Herbaceous‐Shrub Species in the Cerrado.” Plant Ecology 223: 85–97.

[ece371623-bib-0004] * Ban, S. , T. Sakane , and A. Nakagiri . 2015. “Three New Species of *Ophiocordyceps* and Overview of Anamorph Types in the Genus and the Family Ophiocordyceptaceae.” Mycological Progress 14: 1–12.

[ece371623-bib-0005] * Ban, S. , T. Sakane , K. Toyama , and A. Nakagiri . 2009. “Teleomorph‐Anamorph Relationships and Reclassification of *Cordyceps cuboidea* and Its Allied Species.” Mycoscience 50: 261–272.

[ece371623-bib-0006] * Barron, G. L. 1977. “Nematophagous Fungi: A New *Harposporium* Parasitic on Prismatolaimus.” Canadian Journal of Botany 55: 892–895.

[ece371623-bib-0007] * Barron, G. L. 1980. “Fungal Parasites of Rotifers: A New *Tolypocladium* With Underwater Conidiation.” Canadian Journal of Botany 58: 439–442.

[ece371623-bib-0008] Batalla‐Carrera, L. , A. Morton , S. Santamaria , and F. García‐del‐Pino . 2013. “Isolation and Virulence of Entomopathogenic Fungi Against Larvae of Hazelnut Weevil *Curculio nucum* (Coleoptera, Curculionidae) and the Effects of Combining *Metarhizium anisopliae* With Entomopathogenic Nematodes in the Laboratory.” Biocontrol Science and Technology 23: 101–125.

[ece371623-bib-0009] * Binimelis‐Salazar, J. , A. Casanova‐Katny , N. Arnold , et al. 2021. “Diversity and Host Relationships of the *Mycoparasite sepedonium* (Hypocreales, Ascomycota) in Temperate Central Chile.” Microorganisms 9: 2261. 34835387 10.3390/microorganisms9112261 PMC8624339

[ece371623-bib-0010] Butt, T. M. , C. J. Coates , I. M. Dubovskiy , and N. A. Ratcliffe . 2016. “Entomopathogenic Fungi: New Insights Into Host‐Pathogen Interactions.” Advances in Genetics 94: 307–364. 27131329 10.1016/bs.adgen.2016.01.006

[ece371623-bib-0011] Cabrera‐Mora, J. A. , A. W. Guzmán‐Franco , M. T. Santillán‐Galicia , and F. Tamayo‐Mejía . 2019. “Niche Separation of Species of Entomopathogenic Fungi Within the Genera *Metarhizium* and *Beauveria* in Different Cropping Systems in Mexico.” Fungal Ecology 39: 349–355.

[ece371623-bib-0012] * Canassa, F. , F. C. Esteca , R. A. Moral , N. V. Meyling , I. Klingen , and I. Delalibera . 2020. “Root Inoculation of Strawberry With the Entomopathogenic Fungi *Metarhizium robertsii* and *Beauveria bassiana* Reduces Incidence of the Twospotted Spider Mite and Selected Insect Pests and Plant Diseases in the Field.” Journal of Pest Science 93: 261–274.

[ece371623-bib-0013] * Cao, J. , X. Wu , and S. Lin . 2012. “Identification of Fungus *Lecanicillium psalliotae* and Its Colonization in Different Life Stages of *Meloidogyne incognita* .” Scientia Agricultura Sinica 45: 2404–2411.

[ece371623-bib-0014] Ceryngier, P. 2000. “Overwintering of *Coccinella septempunctata* (Coleoptera: Coccinellidae) at Different Altitudes in the Karkonosze Mts, SW Poland.” European Journal of Entomology 97: 323–328.

[ece371623-bib-0015] Chen, L. , W. Huang , H. Wu , et al. 2019. “Contrasting Patterns and Drivers of Soil Fungal Communities in Subtropical Deciduous and Evergreen Broadleaved Forests.” Applied Microbiology and Biotechnology 103: 5421–5433. 31073876 10.1007/s00253-019-09867-z

[ece371623-bib-0016] * Chen, S. , Y. Wang , K. Zhu , and H. Yu . 2021. “Mitogenomics, Phylogeny and Morphology Reveal *Ophiocordyceps pingbianensis* sp. nov., an Entomopathogenic Fungus From China.” Life 11: 686. 34357059 10.3390/life11070686 PMC8305939

[ece371623-bib-0017] Chen, Z. , K. Chen , Y. D. Dai , et al. 2019. “ *Beauveria* Species Diversity in the Gaoligong Mountains of China.” Mycological Progress 18: 933–943.

[ece371623-bib-0018] * Cheng, Y. , J. Yang , T. Li , et al. 2024. “Endosymbiotic Fungal Diversity and Dynamics of the Brown Planthopper Across Developmental Stages, Tissues, and Sexes Revealed Using Circular Consensus Sequencing.” Insects 15: 87. 38392507 10.3390/insects15020087 PMC10889434

[ece371623-bib-0019] Chroňáková, A. , J. Bárta , E. Kaštovská , Z. Urbanová , and T. Picek . 2019. “Spatial Heterogeneity of Belowground Microbial Communities Linked to Peatland Microhabitats With Different Plant Dominants.” FEMS Microbiology Ecology 95: fiz130. 31425589 10.1093/femsec/fiz130 PMC8117459

[ece371623-bib-0020] * Clifton, E. H. , L. A. Castrillo , and A. E. Hajek . 2021. “Discovery of Two Hypocrealean Fungi Infecting Spotted Lanternflies, *Lycorma delicatula*: *Metarhizium pemphigi* and a Novel Species, *Ophiocordyceps delicatula* .” Journal of Invertebrate Pathology 186: 107689. 34774856 10.1016/j.jip.2021.107689

[ece371623-bib-0021] Coates, B. S. , R. L. Hellmich , and L. C. Lewis . 2002. “Allelic Variation of a *Beauveria bassiana* (Ascomycota: Hypocreales) Minisatellite Is Independent of Host Range and Geographic Origin.” Genome 45: 125–132. 11908654 10.1139/g01-132

[ece371623-bib-0022] Corneo, P. E. , A. Pellegrini , L. Cappellin , et al. 2013. “Microbial Community Structure in Vineyard Soils Across Altitudinal Gradients and in Different Seasons.” FEMS Microbiology Ecology 84: 588–602. 23398556 10.1111/1574-6941.12087

[ece371623-bib-0023] * Corrêa, B. , V. da Silveira Duarte , D. M. Silva , G. M. Mascarin , and I. D. Júnior . 2020. “Comparative Analysis of Blastospore Production and Virulence of *Beauveria bassiana* and *Cordyceps fumosorosea* Against Soybean Pests.” BioControl 65: 323–337.

[ece371623-bib-0024] Cota, L. V. , L. A. Maffia , and E. S. G. Mizubuti . 2008. “Brazilian Isolates of *Clonostachys rosea*: Colonization Under Different Temperature and Moisture Conditions and Temporal Dynamics on Strawberry Leaves.” Letters in Applied Microbiology 46: 312–317. 18179592 10.1111/j.1472-765X.2007.02312.x

[ece371623-bib-0025] Dai, L. , L. Qi , Q. Wang , et al. 2011. “Changes in Forest Structure and Composition on Changbai Mountain in Northeast China.” Annals of Forest Science 68: 889–897.

[ece371623-bib-0026] Deaver, N. R. , C. Hesse , C. R. Kuske , and A. Porras‐Alfaro . 2019. “Presence and Distribution of Insect‐Associated and Entomopathogenic Fungi in a Temperate Pine Forest Soil: An Integrated Approach.” Fungal Biology 123: 864–874. 31733729 10.1016/j.funbio.2019.09.006

[ece371623-bib-0027] * Fernandes, C. , A. Casadevall , and T. Gonçalves . 2023. “Mechanisms of *Alternaria* Pathogenesis in Animals and Plants.” FEMS Microbiology Reviews 47: fuad061. 37884396 10.1093/femsre/fuad061

[ece371623-bib-0028] Filotas, M. J. , and A. E. Hajek . 2004. “Influence of Temperature and Moisture on Infection of Forest Tent Caterpillars (Lepidoptera: Lasiocampidae) Exposed to Resting Spores of the Entomopathogenic Fungus *Furia gastropachae* (Zygomycetes: Entomophthorales).” Environmental Entomology 33: 1127–1136.

[ece371623-bib-0029] Fisher, J. J. , S. A. Rehner , and D. J. Bruck . 2011. “Diversity of Rhizosphere Associated Entomopathogenic Fungi of Perennial Herbs, Shrubs and Coniferous Trees.” Journal of Invertebrate Pathology 106: 289–295. 21056569 10.1016/j.jip.2010.11.001

[ece371623-bib-0030] Gandhi, K. J. , D. W. Gilmore , S. A. Katovich , W. J. Mattson , J. R. Spence , and S. J. Seybold . 2007. “Physical Effects of Weather Events on the Abundance and Diversity of Insects in North American Forests.” Environmental Reviews 15: 113–152.

[ece371623-bib-0031] Garrido‐Jurado, I. , J. Torrent , V. Barrón , A. Corpas , and E. Quesada‐Moraga . 2011. “Soil Properties Affect the Availability, Movement, and Virulence of Entomopathogenic Fungi Conidia Against Puparia of *Ceratitis capitata* (Diptera: Tephritidae).” Biological Control 58: 277–285.

[ece371623-bib-0032] Geml, J. , N. Pastor , L. Fernandez , et al. 2014. “Large‐Scale Fungal Diversity Assessment in the Andean Yungas Forests Reveals Strong Community Turnover Among Forest Types Along an Altitudinal Gradient.” Molecular Ecology 23: 2452–2472. 24762095 10.1111/mec.12765

[ece371623-bib-0033] * Glare, T. R. , S. D. Reay , T. L. Nelson , and R. Moore . 2008. “ *Beauveria caledonica* Is a Naturally Occurring Pathogen of Forest Beetles.” Mycological Research 112: 352–360. 18308525 10.1016/j.mycres.2007.10.015

[ece371623-bib-0034] Gleason, F. H. , C. N. Daynes , and P. A. McGee . 2010. “Some Zoosporic Fungi Can Grow and Survive Within a Wide pH Range.” Fungal Ecology 3: 31–37.

[ece371623-bib-0036] Grum‐Grzhimaylo, A. A. , M. L. Georgieva , S. A. Bondarenko , A. J. Debets , and E. N. Bilanenko . 2016. “On the Diversity of Fungi From Soda Soils.” Fungal Diversity 76: 27–74.

[ece371623-bib-0037] Hallouti, A. , M. Ait Hamza , A. Zahidi , et al. 2020. “Diversity of Entomopathogenic Fungi Associated With Mediterranean Fruit Fly ( *Ceratitis capitata* (Diptera: Tephritidae)) in Moroccan Argan Forests and Nearby Area: Impact of Soil Factors on Their Distribution.” BMC Ecology 20: 1–13. 33234114 10.1186/s12898-020-00334-2 PMC7684748

[ece371623-bib-0038] Hao, Z. , S. Guo , and J. Ye . 2003. “Canonical Correspodence Analysis on Relationship of Woody Plants With Environments on the Northern Slop of Changbai Mountain.” Acta Phytoecologica Sinica 27: 733–741.

[ece371623-bib-0039] * Haritakun, R. , M. Sappan , R. Suvannakad , K. Tasanathai , and M. Isaka . 2010. “An Antimycobacterial Cyclodepsipeptide From the Entomopathogenic Fungus *Ophiocordyceps communis* BCC 16475.” Journal of Natural Products 73: 75–78. 20028029 10.1021/np900520b

[ece371623-bib-0040] Hartmann, M. , and J. Six . 2023. “Soil Structure and Microbiome Functions in Agroecosystems.” Nature Reviews Earth and Environment 4: 4–18.

[ece371623-bib-0041] Hesketh, H. , H. E. Roy , J. Eilenberg , J. K. Pell , and R. S. Hails . 2010. “Challenges in Modelling Complexity of Fungal Entomopathogens in Semi‐Natural Populations of Insects.” BioControl 55: 55–73.

[ece371623-bib-0042] * Hywel‐Jones, N. 1995. “ *Cordyceps sphecocephala* and a *Hymenostilbe* sp. Infecting Wasps and Bees in Thailand.” Mycological Research 99: 154–158.

[ece371623-bib-0043] Islam, W. , M. Adnan , A. Shabbir , et al. 2021. “Insect‐Fungal‐Interactions: A Detailed Review on Entomopathogenic Fungi Pathogenicity to Combat Insect Pests.” Microbial Pathogenesis 159: 105122. 34352375 10.1016/j.micpath.2021.105122

[ece371623-bib-0044] * Iwasaki, H. , T. Tokiwa , M. Shiina , et al. 2019. “ *Metarhizium aciculare* sp. nov. for Euvesperins A and B Producing *Metarhizium* Strains.” Mycoscience 60: 313–318.

[ece371623-bib-0045] Jaber, L. R. , and B. H. Ownley . 2018. “Can We Use Entomopathogenic Fungi as Endophytes for Dual Biological Control of Insect Pests and Plant Pathogens?” Biological Control 116: 36–45.

[ece371623-bib-0046] Jackson, M. A. , C. A. Dunlap , and S. T. Jaronski . 2010. “Ecological Considerations in Producing and Formulating Fungal Entomopathogens for Use in Insect Biocontrol.” BioControl 55: 129–145.

[ece371623-bib-0047] Jin, Y. , J. Xu , H. He , et al. 2021. “Effects of Catastrophic Wind Disturbance on Formation of Forest Patch Mosaic Structure on the Western and Southern Slopes of Changbai Mountain.” Forest Ecology and Management 481: 118746.

[ece371623-bib-0048] Kreutz, J. , G. Zimmermann , and O. Vaupel . 2004. “Horizontal Transmission of the Entomopathogenic Fungus *Beauveria bassiana* Among the Spruce Bark Beetle, *Ips typographus* (Col., Scolytidae) in the Laboratory and Under Field Conditions.” Biocontrol Science and Technology 14: 837–848.

[ece371623-bib-0049] Kryukov, V. Y. , O. N. Yaroslavtseva , I. M. Dubovskiy , M. V. Tyurin , N. A. Kryukova , and V. V. Glupov . 2014. “Insecticidal and Immunosuppressive Effect of Ascomycete *Cordyceps militaris* on the Larvae of the Colorado Potato Beetle *Leptinotarsa decemlineata* .” Biology Bulletin 41: 276–283. 25731041

[ece371623-bib-0050] Kumar, C. S. , T. K. Jacob , S. Devasahayam , S. D'Silva , and C. Geethu . 2022. “Field Evaluation of *Lecanicillium psalliotae* and Development of an Integrated Pest Management Strategy Against *Sciothrips cardamomi* .” Biological Control 165: 104822.

[ece371623-bib-0051] Lal, R. 2020. “Soil Organic Matter and Water Retention.” Agronomy Journal 112: 3265–3277.

[ece371623-bib-0052] * Langenfeld, A. , A. Blond , S. Gueye , et al. 2011. “Insecticidal Cyclodepsipeptides From *Beauveria felina* .” Journal of Natural Products 74: 825–830. 21438588 10.1021/np100890n

[ece371623-bib-0053] Li, W. Q. , Y. Huang , F. Chen , et al. 2021. “Mixing With Broad‐Leaved Trees Shapes the Rhizosphere Soil Fungal Communities of Coniferous Tree Species in Subtropical Forests.” Forest Ecology and Management 480: 118664.

[ece371623-bib-0054] * Liu, H. , M. Skinner , B. L. Parker , and M. Brownbridge . 2002. “Pathogenicity of *Beauveria bassiana*, *Metarhizium anisopliae* (Deuteromycotina: Hyphomycetes), and Other Entomopathogenic Fungi Against *Lygus lineolaris* (Hemiptera: Miridae).” Journal of Economic Entomology 95: 675–681. 12216806 10.1603/0022-0493-95.4.675

[ece371623-bib-0055] Lopes, R. B. , D. A. de Souza , P. W. Inglis , and M. Faria . 2023. “Diversity of Anamorphic *Cordyceps* (Formerly Isaria) Isolated From Brazilian Agricultural Sites.” Journal of Invertebrate Pathology 200: 107956. 37356705 10.1016/j.jip.2023.107956

[ece371623-bib-0056] López‐Lima, D. , D. Alarcón‐Utrera , J. Á. Ordáz‐Meléndez , L. Villain , and G. Carrión . 2023. “ *Metarhizium carneum* Formulations: A Promising New Biological Control to Be Incorporated in the Integrated Management of *Meloidogyne enterolobii* on Tomato Plants.” Plants 12: 3431. 37836171 10.3390/plants12193431 PMC10574380

[ece371623-bib-0057] * Luangsa‐Ard, J. J. , R. Ridkaew , S. Mongkolsamrit , K. Tasanathai , and N. L. Hywel‐Jones . 2010. “ *Ophiocordyceps barnesii* and Its Relationship to Other Melolonthid Pathogens With Dark Stromata.” Fungal Biology 114: 739–745. 20943183 10.1016/j.funbio.2010.06.007

[ece371623-bib-0058] Masoudi, A. , J. L. Koprowski , U. R. Bhattarai , and D. Wang . 2018. “Elevational Distribution and Morphological Attributes of the Entomopathogenic Fungi From Forests of the Qinling Mountains in China.” Applied Microbiology and Biotechnology 102: 1483–1499. 29189901 10.1007/s00253-017-8651-4

[ece371623-bib-0059] * Mauchline, N. , I. Hallett , G. Hill , and S. Casonato . 2011. “Process of Infection of Armored Scale Insects (Diaspididae) by an Entomopathogenic *Cosmospora* sp.” Journal of Invertebrate Pathology 108: 46–51. 21771597 10.1016/j.jip.2011.07.002

[ece371623-bib-0060] Mayerhofer, J. , S. Eckard , M. Hartmann , et al. 2017. “Assessing Effects of the Entomopathogenic Fungus *Metarhizium brunneum* on Soil Microbial Communities in *Agriotes* spp. Biological Pest Control.” FEMS Microbiology Ecology 93: fix117. 28961941 10.1093/femsec/fix117 PMC5812499

[ece371623-bib-0061] McKinnon, A. C. , T. R. Glare , H. J. Ridgway , et al. 2018. “Detection of the Entomopathogenic Fungus *Beauveria bassiana* in the Rhizosphere of Wound‐Stressed *Zea mays* Plants.” Frontiers in Microbiology 9: 1161. 29942287 10.3389/fmicb.2018.01161 PMC6004820

[ece371623-bib-0062] Moloinyane, S. , P. Addison , K. A. Achiano , and F. Nchu . 2020. “Association Between Chemical Properties of Vineyard Soils and Occurrence of Entomopathogenic Fungi Causing Different Levels of Mortality in *Planococcus ficus* .” BioControl 65: 197–209.

[ece371623-bib-0063] Moreno‐Gavíra, A. , V. Huertas , F. Diánez , B. Sánchez‐Montesinos , and M. Santos . 2020. “ *Paecilomyces* and Its Importance in the Biological Control of Agricultural Pests and Diseases.” Plants 9: 1746. 33321854 10.3390/plants9121746 PMC7763231

[ece371623-bib-0064] Mueller‐Dombois, D. , and H. Ellenberg . 1974. Aims and Methods of Vegetation Ecology. John Wiley & Sons, Inc.

[ece371623-bib-0065] Naranjo‐Ortiz, M. A. , and T. Gabaldón . 2019. “Fungal Evolution: Major Ecological Adaptations and Evolutionary Transitions.” Biological Reviews 94: 1443–1476. 31021528 10.1111/brv.12510 PMC6850671

[ece371623-bib-0066] Nazir, R. , J. A. Warmink , H. Boersma , and J. D. Van Elsas . 2009. “Mechanisms That Promote Bacterial Fitness in Fungal‐Affected Soil Microhabitats.” FEMS Microbiology Ecology 71: 169–185. 20002182 10.1111/j.1574-6941.2009.00807.x

[ece371623-bib-0067] Neto, J. A. C. , L. C. Leal , and F. B. Baccaro . 2019. “Temporal and Spatial Gradients of Humidity Shape the Occurrence and the Behavioral Manipulation of Ants Infected by Entomopathogenic Fungi in Central Amazon.” Fungal Ecology 42: 100871.

[ece371623-bib-0068] * Oliveira, I. , J. A. Pereira , T. Lino‐Neto , A. Bento , and P. Baptista . 2012. “Fungal Diversity Associated to the Olive Moth, *Prays oleae* Bernard: A Survey for Potential Entomopathogenic Fungi.” Microbial Ecology 63: 964–974. 21994034 10.1007/s00248-011-9955-z

[ece371623-bib-0069] Padmavathi, J. , K. Uma Devi , and C. Uma Maheswara Rao . 2003. “The Optimum and Tolerance pH Range Is Correlated to Colonial Morphology in Isolates of the Entomopathogenic Fungus *Beauveria bassiana*—A Potential Biopesticide.” World Journal of Microbiology and Biotechnology 19: 469–477.

[ece371623-bib-0070] Papudeshi, B. , D. B. Rusch , D. VanInsberghe , C. M. Lively , R. A. Edwards , and F. Bashey . 2023. “Host Association and Spatial Proximity Shape but Do Not Constrain Population Structure in the Mutualistic Symbiont *Xenorhabdus bovienii* .” MBio 14: e00434–23. 37154562 10.1128/mbio.00434-23 PMC10306267

[ece371623-bib-0071] Peng, C. Y. , X. Zhou , and H. K. Kaya . 2002. “Virulence and Site of Infection of the Fungus, *Hirsutella thompsonii*, to the Honey Bee Ectoparasitic Mite, *Varroa destructor* .” Journal of Invertebrate Pathology 81: 185–195. 12507488 10.1016/s0022-2011(02)00188-x

[ece371623-bib-0201] Peng, T. L. , S. A. Syazwan , and S. H. Lee . 2019. Soil‐Borne Entomopathogenic Bacteria and Fungi. In *Microbes for Sustainable Insect Pest Management* , edited by M. Khan, and W. Ahmad. Sustainability in Plant and Crop Protection. Springer.

[ece371623-bib-0072] Pilarska, D. , M. McManus , P. Pilarski , G. Georgiev , P. Mirchev , and A. Linde . 2006. “Monitoring the Establishment and Prevalence of the Fungal Entomopathogen *Entomophaga maimaiga* in Two *Lymantria dispar* L. Populations in Bulgaria.” Journal of Pest Science 79: 63–67.

[ece371623-bib-0073] Popa, V. , E. Deziel , R. Lavallée , E. Bauce , and C. Guertin . 2012. “The Complex Symbiotic Relationships of Bark Beetles With Microorganisms: A Potential Practical Approach for Biological Control in Forestry.” Pest Management Science 68: 963–975. 22566204 10.1002/ps.3307

[ece371623-bib-0074] Przybył, K. , P. Karolewski , J. Oleksyn , A. Łabędzki , and P. B. Reich . 2008. “Fungal Diversity of Norway Spruce Litter: Effects of Site Conditions and Premature Leaf Fall Caused by Bark Beetle Outbreak.” Microbial Ecology 56: 332–340. 18095016 10.1007/s00248-007-9350-y

[ece371623-bib-0075] Qiu, H. , E. G. Fox , C. Qin , et al. 2023. “First Record of *Fusarium concentricum* (Hypocreales: Hypocreaceae) Isolated From the Moth *Polychrosis cunninhamiacola* (Lepidoptera: Tortricidae) as an Entomopathogenic Fungus.” Journal of Insect Science 23, no. 2: 2. 10.1093/jisesa/iead008 PMC10011878 36916278

[ece371623-bib-0076] Quesada‐Moraga, E. , J. A. Navas‐Cortés , E. A. Maranhao , A. Ortiz‐Urquiza , and C. Santiago‐Álvarez . 2007. “Factors Affecting the Occurrence and Distribution of Entomopathogenic Fungi in Natural and Cultivated Soils.” Mycological Research 111: 947–966. 17766099 10.1016/j.mycres.2007.06.006

[ece371623-bib-0077] Raja, R. K. , A. Arun , M. Touray , et al. 2021. “Antagonists and Defense Mechanisms of Entomopathogenic Nematodes and Their Mutualistic Bacteria.” Biological Control 152: 104452.

[ece371623-bib-0078] Rajula, J. , A. Rahman , and P. Krutmuang . 2020. “Entomopathogenic Fungi in Southeast Asia and Africa and Their Possible Adoption in Biological Control.” Biological Control 151: 104399.

[ece371623-bib-0079] Rangel, D. E. , G. U. Braga , É. K. Fernandes , C. A. Keyser , J. E. Hallsworth , and D. W. Roberts . 2015. “Stress Tolerance and Virulence of Insect‐Pathogenic Fungi Are Determined by Environmental Conditions During Conidial Formation.” Current Genetics 61: 383–404. 25791499 10.1007/s00294-015-0477-y

[ece371623-bib-0080] * Rodrigues, J. , L. F. Rocha , J. M. Martinez , C. Montalva , R. A. Humber , and C. Luz . 2022. “ *Clonostachys* spp., Natural Mosquito Antagonists, and Their Prospects for Biological Control of *Aedes aegypti* .” Parasitology Research 121: 2979–2984. 35994116 10.1007/s00436-022-07630-4

[ece371623-bib-0081] Roy, H. E. , D. C. Steinkraus , J. Eilenberg , A. E. Hajek , and J. K. Pell . 2006. “Bizarre Interactions and Endgames: Entomopathogenic Fungi and Their Arthropod Hosts.” Annual Review of Entomology 51: 331–357. 10.1146/annurev.ento.51.110104.150941 16332215

[ece371623-bib-0082] * Ruiz‐Jiménez, A. L. , E. Ruiz‐Sánchez , G. Heredia , et al. 2019. “Identification of Insect‐Deterrent Metabolites From *Acremonium masseei* Strain CICY026, a Saprophytic Fungus From a Sinkhole in Yucatán.” Microorganisms 7: 712. 31861143 10.3390/microorganisms7120712 PMC6955848

[ece371623-bib-0083] * Sasaki, F. , T. Miyamoto , Y. Tamai , and T. Yajima . 2007. “Note on *Cordyceps brongniartii* Shimazu Collected From the Wild in Japan.” Mycoscience 48: 312–315.

[ece371623-bib-0084] * Sasaki, F. , T. Miyamoto , A. Yamamoto , Y. Tamai , and T. Yajima . 2012. “Relationship Between Intraspecific Variations and Host Insects of *Ophiocordyceps nutans* Collected in Japan.” Mycoscience 53: 85–91.

[ece371623-bib-0085] Scheepmaker, J. W. A. , and T. M. Butt . 2010. “Natural and Released Inoculum Levels of Entomopathogenic Fungal Biocontrol Agents in Soil in Relation to Risk Assessment and in Accordance With EU Regulations.” Biocontrol Science and Technology 20: 503–552.

[ece371623-bib-0086] Seguel, A. , J. R. Cumming , K. Klugh‐Stewart , P. Cornejo , and F. Borie . 2013. “The Role of Carbuncular Mycorrhizas in Decreasing Aluminium Phytotoxicity in Acidic Soils: A Review.” Mycorrhiza 23: 167–183. 23328806 10.1007/s00572-013-0479-x

[ece371623-bib-0087] * Seye, F. , T. Bawin , S. Boukraa , et al. 2014. “Effect of Entomopathogenic *Aspergillus* Strains Against the Pea Aphid, *Acyrthosiphon pisum* (Hemiptera: Aphididae).” Applied Entomology and Zoology 49: 453–458.

[ece371623-bib-0088] * Sharma, L. , and G. Marques . 2018. “ *Fusarium*, an Entomopathogen‐A Myth or Reality?” Pathogens 7: 93. 30487454 10.3390/pathogens7040093 PMC6314043

[ece371623-bib-0089] * Shrestha, B. , W. Zhang , Y. Zhang , and X. Liu . 2012. “The Medicinal Fungus *Cordyceps militaris*: Research and Development.” Mycological Progress 11: 599–614.

[ece371623-bib-0090] Skrzecz, I. , A. Sierpińska , and D. Tumialis . 2024. “Entomopathogens in the Integrated Management of Forest Insects: From Science to Practice.” Pest Management Science 80: 2503–2514. 37983918 10.1002/ps.7871

[ece371623-bib-0092] Sumida, A. , T. Miyaura , and H. Torii . 2013. “Relationships of Tree Height and Diameter at Breast Height Revisited: Analyses of Stem Growth Using 20‐Year Data of an Even‐Aged *Chamaecyparis obtusa* Stand.” Tree Physiology 33: 106–118. 23303367 10.1093/treephys/tps127 PMC3556985

[ece371623-bib-0093] * Sung, G. H. , and J. W. Spatafora . 2004. “ *Cordyceps cardinalis* sp. nov., a New Species of *Cordyceps* With an East Asian‐Eastern North American Distribution.” Mycologia 96: 658–666. 21148886

[ece371623-bib-0094] Tedersoo, L. , M. Bahram , S. Põlme , et al. 2014. “Global Diversity and Geography of Soil Fungi.” Science 346: 1256688. 25430773 10.1126/science.1256688

[ece371623-bib-0095] * Uday, K. , and B. Bhagawati . 2022. “Parasitism of *Meloidogyne incognita* Eggs by Native Fungi of Assam.” Ecology, Environment and Conservation 28: S149–S153.

[ece371623-bib-0096] * Upadhyay, V. , D. Rai , M. Rana , P. Mehra , and A. K. Pandey . 2014. “ *Verticillium lecani* (Zimm.): A Potential Entomopathogenic Fungus.” International Journal of Agriculture, Environment and Biotechnology 7: 719–727.

[ece371623-bib-0097] Uzman, D. , J. Pliester , I. Leyer , M. H. Entling , and A. Reineke . 2019. “Drivers of Entomopathogenic Fungi Presence in Organic and Conventional Vineyard Soils.” Applied Soil Ecology 133: 89–97.

[ece371623-bib-0098] Vukicevich, E. , D. T. Lowery , J. A. Bennett , and M. Hart . 2019. “Influence of Groundcover Vegetation, Soil Physicochemical Properties, and Irrigation Practices on Soil Fungi in Semi‐Arid Vineyards.” Frontiers in Ecology and Evolution 7: 118.

[ece371623-bib-0099] Wakil, W. , M. U. Ghazanfar , and M. Yasin . 2014. “Naturally Occurring Entomopathogenic Fungi Infecting Stored Grain Insect Species in Punjab, Pakistan.” Journal of Insect Science 14: 182. 25480970 10.1093/jisesa/ieu044 PMC5634054

[ece371623-bib-0100] Wang, J. B. , R. S. Leger , and C. Wang . 2016. “Chapter Three—Advances in Genomics of Insect Pathogenic Fungi, in Advances.” In Genetics, edited by B. Lovett and R. J. St. Leger , 67–105. Elsevier. 10.1016/bs.adgen.2016.01.002 27131323

[ece371623-bib-0101] Wang, Y. , Y. Liu , D. Tang , Y. Wang , A. E. Adams , and H. Yu . 2022. “ *Tolypocladium reniformisporum* sp. Nov. and *Tolypocladium cylindrosporum* (Ophiocordycipitaceae, Hypocreales) Co‐Occurring on *Ophiocordyceps sinensis* .” Mycological Progress 21: 199–214.

[ece371623-bib-0102] Wang, Y. , D. Tang , D. Duan , Y. Wang , and H. Yu . 2020. “Morphology, Molecular Characterization, and Virulence of *Beauveria pseudobassiana* Isolated From Different Hosts.” Journal of Invertebrate Pathology 172: 107333. 32001215 10.1016/j.jip.2020.107333

[ece371623-bib-0103] * Wang, Y. B. , H. Yu , Y. Dai , et al. 2015. “ *Polycephalomyces agaricus*, a New Hyperparasite of *Ophiocordyceps* sp. Infecting Melolonthid Larvae in Southwestern China.” Mycological Progress 14: 1–9.

[ece371623-bib-0104] * Whyte, A. C. , J. B. Gloer , D. T. Wicklow , and P. F. Dowd . 1996. “Sclerotiamide: A New Member of the Paraherquamide Class With Potent Antiinsectan Activity From the Sclerotia of *Aspergillus sclerotiorum* .” Journal of Natural Products 59: 1093–1095. 8946752 10.1021/np960607m

[ece371623-bib-0105] Yu, G. , Z. Chen , S. Piao , et al. 2014. “High Carbon Dioxide Uptake by Subtropical Forest Ecosystems in the East Asian Monsoon Region.” Proceedings of the National Academy of Sciences of the United States of America 111: 4910–4915. 24639529 10.1073/pnas.1317065111 PMC3977309

[ece371623-bib-0106] Zembrzuski, D. , D. A. Woller , S. Jaronski , et al. 2023. “Understanding How Diet and Temperature Affect Survival and Subsequent Sporulation in a Major Rangeland Grasshopper Pest, *Melanoplus sanguinipes* , Infected With the Entomopathogenic Fungus, *Metarhizium Robertsii* .” Biological Control 183: 105268.

[ece371623-bib-0107] Zha, L. , V. Y. Kryukov , J. Ding , R. Jeewon , and P. Chomnunti . 2021. “Novel Taxa and Species Diversity of *Cordyceps* Sensu Lato (Hypocreales, Ascomycota) Developing on Wireworms (Elateroidea and Tenebrionoidea, Coleoptera).” MycoKeys 78: 79–117. 33854402 10.3897/mycokeys.78.61836 PMC8021543

[ece371623-bib-0108] * Zhang, X. , Q. Hu , and Q. Weng . 2019. “Secondary Metabolites (SMs) of *Isaria cicadae* and *Isaria tenuipes* .” RSC Advances 9: 172–184. 10.1039/c8ra09039d PMC9059538 35521576

[ece371623-bib-0109] Zhang, Y. , S. Zhang , Y. Li , et al. 2014. “Phylogeography and Evolution of a Fungal–Insect Association on the Tibetan Plateau.” Molecular Ecology 23: 5337–5355. 25263531 10.1111/mec.12940

[ece371623-bib-0110] Zheng, J. , J. Shi , and D. Wang . 2024. “Diversity of Soil Fungi and Entomopathogenic Fungi in Subtropical Mountain Forest in Southwest China.” Environmental Microbiology Reports 16: e13267. 38943366 10.1111/1758-2229.13267 PMC11213981

[ece371623-bib-0111] Zhou, Y. , F. Meng , B. Ochieng , et al. 2024. “Climate and Environmental Variables Drive Stream Biofilm Bacterial and Fungal Diversity on Tropical Mountainsides.” Microbial Ecology 87: 28. 38182675 10.1007/s00248-023-02335-2 PMC13496561

