# Supplementary figures and images for: Vertical Zonal Distribution Patterns of Entomopathogenic Fungi in the Changbai Mountain
Source: Ecol Evol. 2025 Jul 1;15(7):e71623. doi: 10.1002/ece3.71623 (PMC12213609; doi:10.1002/ece3.71623)

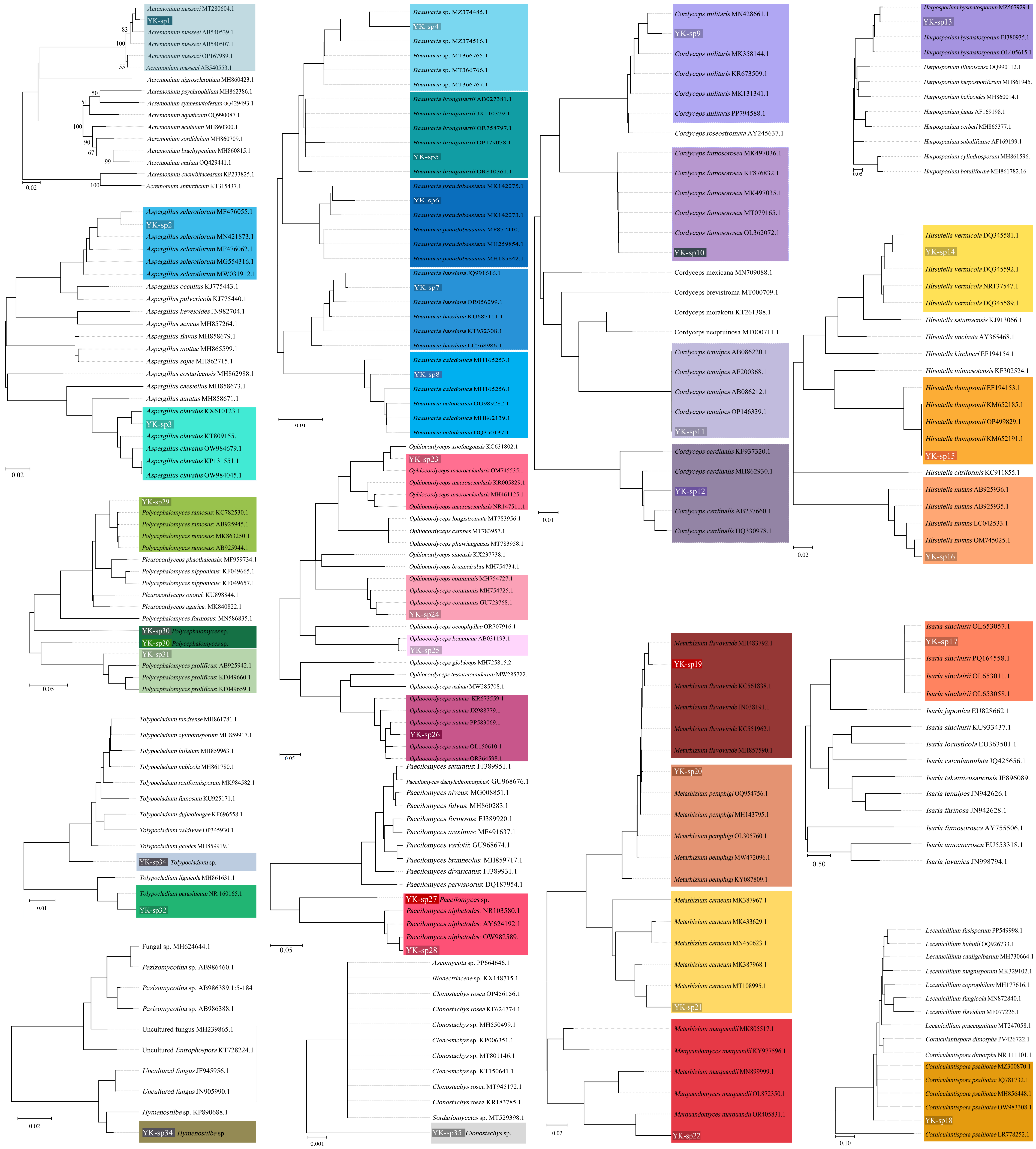

Supplement: Supplementary file 1 — Figure S1. Phylogenetic tree of EPF from Changbai Mountain. [file ECE3-15-e71623-s004.png]

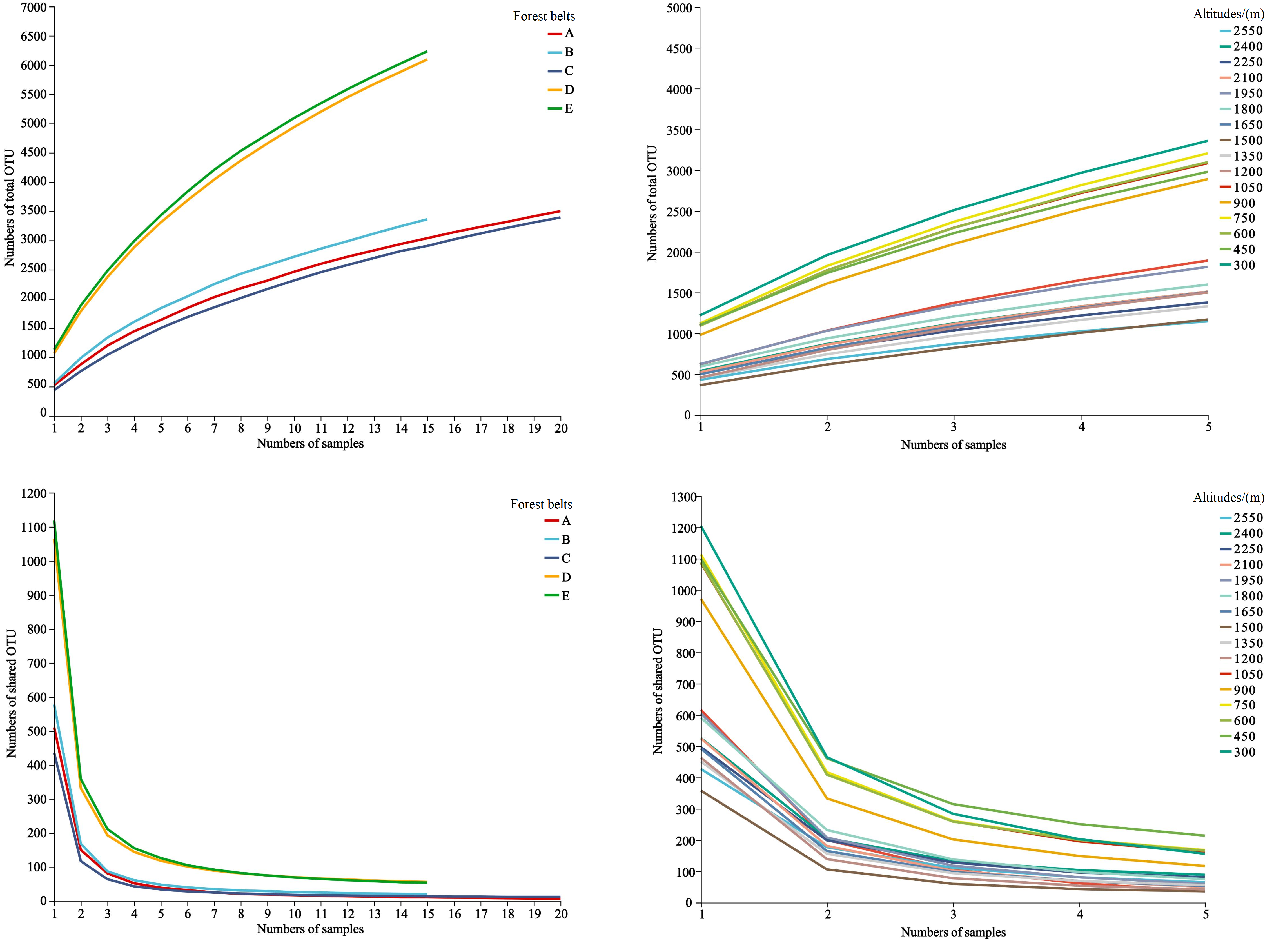

Supplement: Supplementary file 2 — Figure S2. Pan_OTU and Core_OTU statistics of EPF communities across different forest belts and altitudes. [file ECE3-15-e71623-s002.png]
