# Supplementary material for: Vertical Zonal Distribution Patterns of Entomopathogenic Fungi in the Changbai Mountain
Source: Ecol Evol. 2025 Jul 1;15(7):e71623. doi: 10.1002/ece3.71623 (PMC12213609; doi:10.1002/ece3.71623)
Supplement: Supplementary file 3 — Table S1. Species composition of EPF in Changbai Mountain. Note: Species identified as EPF are referenced from relevant publications. An asterisk (*) indicates that when the number of unidentified species in a genus equals 0, the genus is considered absent in Changbai Mountain and is excluded from subsequent analysis. [file ECE3-15-e71623-s003.docx]

Table S1 Species composition of entomopathogenic fungi in Changbai Mountain

| Kingdom | Phylum | Class | Order | Family | Entomopathogenic fungal genera | No. of identified species | No. of unidentified species | Entomopathogenic fungal species |
| --- | --- | --- | --- | --- | --- | --- | --- | --- |
| Fungi | Ascomycota | Dothideomycetes | Pleosporales | Pleosporaceae | *Alternaria* (Fernandes et al., 2023) | 0 | 1 |  |
|  |  |  | Capnodiales | Cladosporiaceae | *Cladosporium* (Chen et al., 2021) | 2 | 0 | ***** |
|  |  | Eurotiomycetes | Eurotiales | Aspergillaceae | *Aspergillus* | 10 | 7 | *Aspergillus clavatu* (Seye et al., 2014) |
|  |  |  |  |  |  |  |  | *Aspergillus sclerotiorum* (Whyte et al., 1996) |
|  |  |  |  |  | *Penicillium* (Oliveira, 2012) | 22 | 17 |  |
|  |  |  |  |  | *Paecilomyces* | 2 | 0 | *Paecilomyces niphetodes* (Uday et al., 2022) |
|  |  |  |  |  |  |  |  | *Paecilomyces* sp. (Moreno-Gavíra et al., 2020) |
|  |  |  |  | Trichocomaceae | *Sepedonium* (Binimelis-Salazar et al., 2004) | 1 | 0 | ***** |
|  |  | Sordariomycetes | Hypocreales | Hypocreaceae | *Sepedonium* (Binimelis-Salazar et al., 2004) | 1 | 0 | ***** |
|  |  |  |  | Bionectriaceae | *Clonostachys* | 11 | 2 | *Clonostachys* sp. (Rodrigues et al., 2022) |
|  |  |  |  |  | *Acremonium* | 3 | 0 | *Acremonium masseei* (Ruiz-Jiménez et al., 2019) |
|  |  |  |  | Clavicipitaceae | *Metarhizium* | 13 | 2 | *Metarhizium carneum* (López-Lima et al., 2023) |
|  |  |  |  |  |  |  |  | *Metarhizium marquandii* (Iwasaki et al., 2019) |
|  |  |  |  |  |  |  |  | *Metarhizium pemphigi* (Clifton et al., 2021)  NCBI accession number: PV683227 |
|  |  |  |  |  |  |  |  | *Metarhizium flavoviride* (Xu et al., 2016)  NCBI accession number: PV683067 |
|  |  |  |  |  | *Pochonia* (Chen et al., 2021) | 1 | 2 |  |
|  |  |  |  | Cordycipitaceae | *Beauveria* | 5 | 0 | *Beauveria bassiana* (Canassa et al., 2020)  NCBI accession number: PV683252; PV683253; PV683255; PV683332; PV683344; PV683345; PV683474; PV683565; PV684017 |
|  |  |  |  |  |  |  |  | *Beauveria caledonica* (Glare et al., 2008) |
|  |  |  |  |  |  |  |  | *Beauveria* sp. (Langenfeld et al., 2011)  NCBI accession number: PV683346; PV683386; PV683383 |
|  |  |  |  |  |  |  |  | *Beauveria pseudobassiana* (Wang et al., 2020)  NCBI accession number: PV683388; PV683433; PV683434 |
|  |  |  |  |  |  |  |  | *Beauveria brongniartii* (Sasaki et al., 2007)  NCBI accession number: PV683288; PV683298; PV683331 |
|  |  |  |  |  | *Cordyceps* | 5 | 1 | *Cordyceps cardinalis* (Sung et al., 2004) |
|  |  |  |  |  |  |  |  | *Cordyceps militaris* (Shrestha et al., 2012)  NCBI accession number: PV683235; PV683236; PV683239 |
|  |  |  |  |  |  |  |  | *Cordyceps tenuipes* (Lopes et al., 2023) |
|  |  |  |  |  |  |  |  | *Cordyceps fumosorosea* (Corrêa et al., 2020)  NCBI accession number: PV683261; PV683262; PV683287 |
|  |  |  |  |  | *Isaria* | 1 | 0 | *Isaria sinclairii* (Zhang et al., 2019) |
|  |  |  |  |  | *Lecanicillium* | 5 | 3 | *Lecanicillium psalliotae* (Cao et al., 2012) |
|  |  |  |  | Nectriaceae | *Verticillium* (Upadhyay, 2014) | 2 | 0 | ***** |
|  |  |  |  |  | *Fusarium* (Sharma et al., 2018) | 3 | 10 |  |
|  |  |  |  |  | *Mariannaea* (Liu et al., 2002) | 2 | 6 |  |
|  |  |  |  |  | *Nectria* (Mauchline et al., 2011) | 1 | 0 | ***** |
|  |  |  |  | Ophiocordycipitaceae | *Harposporium* | 1 | 0 | *Harposporium bysmatosporum* (Barron et al., 1977) |
|  |  |  |  |  | *Hirsutella* | 3 | 2 | *Hirsutella nutans* (Peng et al., 2002) |
|  |  |  |  |  |  |  |  | *Hirsutella thompsonii* (Peng et al., 2002) |
|  |  |  |  |  |  |  |  | *Hirsutella vermicola* (Chen et al., 2021) |
|  |  |  |  |  | *Hymenostilbe* | 4 | 0 | *Hymenostilbe* sp. (Hywel-Jones et al., 1995) |
|  |  |  |  |  | *Ophiocordyceps* | 4 | 2 | *Ophiocordyceps communis* (Haritakun et al., 2010) |
|  |  |  |  |  |  |  |  | *Ophiocordyceps konnoana* (Luangsa-Ard et al., 2010) |
|  |  |  |  |  |  |  |  | *Ophiocordyceps macroacicularis* (Ban et al., 2015) |
|  |  |  |  |  |  |  |  | *Ophiocordyceps nutans* (Sasaki et al., 2012) |
|  |  |  |  |  | *Polycephalomyces* | 4 | 2 | *Polycephalomyces prolificus* (Cheng et al., 2024) |
|  |  |  |  |  |  |  |  | *Polycephalomyces ramosus* (Wang et al., 2015) |
|  |  |  |  |  |  |  |  | *Polcephalomyces* sp. (Ban et al., 2009) |
|  |  |  |  |  | *Purpureocillium* (Chen et al., 2021) | 0 | 1 |  |
|  |  |  |  |  | *Tolypocladium* | 7 | 6 | *Tolypocladium parasiticum* (Barron et al., 1980) |
|  |  |  |  |  |  |  |  | *Tolypocladium* sp. (Ruiz-Jiménez et al., 2019) |
