# Supplementary material for: Vertical Zonal Distribution Patterns of Entomopathogenic Fungi in the Changbai Mountain
Source: Ecol Evol. 2025 Jul 1;15(7):e71623. doi: 10.1002/ece3.71623 (PMC12213609; doi:10.1002/ece3.71623)
Supplement: Supplementary file 4 — Table S2. T‐test analysis of diversity index of entomopathogenic fungi at different altitudes. [file ECE3-15-e71623-s001.docx]

TableS2 T-test analysis of the diversity index of entomopathogenic fungi across different altitudes

| **Estimators / m** | **sobs** | | **shannon** | | **chao** | |
| --- | --- | --- | --- | --- | --- | --- |
|  | Mean | sd | Mean | sd | Mean | sd |
| 2550 | 0.400 | 0.548 | 0.000 | 0.000 | 0.400 | 0.548 |
| 2400 | 0.800 | 0.447 | 0.000 | 0.000 | 0.800 | 0.447 |
| 2250 | 1.000 | 1.000 | 0.252 | 0.346 | 1.000 | 1.000 |
| 2100 | 2.000 | 1.581 | 0.411 | 0.399 | 2.000 | 1.581 |
| 1950 | 1.000 | 0.707 | 0.020 | 0.044 | 1.000 | 0.707 |
| 1800 | 2.600 | 2.510 | 0.398 | 0.539 | 2.700 | 2.729 |
| 1650 | 1.400 | 1.517 | 0.267 | 0.419 | 1.400 | 1.517 |
| 1500 | 1.200 | 0.447 | 0.139 | 0.310 | 1.400 | 0.894 |
| 1350 | 2.000 | 1.225 | 0.419 | 0.342 | 2.200 | 1.483 |
| 1200 | 1.800 | 0.837 | 0.339 | 0.371 | 1.800 | 0.837 |
| 1050 | 5.200 | 2.588 | 0.636 | 0.425 | 5.400 | 2.793 |
| 900 | 3.800 | 1.095 | 0.733 | 0.308 | 3.800 | 1.095 |
| 750 | 7.000 | 1.225 | 0.951 | 0.319 | 7.300 | 1.304 |
| 600 | 8.400 | 1.673 | 1.088 | 0.222 | 8.767 | 1.507 |
| 450 | 8.400 | 0.894 | 1.396 | 0.236 | 8.400 | 0.894 |
| 300 | 8.000 | 1.732 | 1.405 | 0.402 | 8.000 | 1.732 |
| *P*_value | <0.001 | | <0.001 | | <0.001 | |
| *P*_adjust | <0.001 | | <0.001 | | <0.001 | |
| Statistic_F | 59.7771 | | 54.8798 | | 59.0112 | |
